# Electrochemical Detection of Drugs via a Supramolecular Cucurbit[7]uril-Based Indicator Displacement Assay

**DOI:** 10.1021/acssensors.3c00008

**Published:** 2023-06-20

**Authors:** Nilima Manoj Kumar, Patrick Gruhs, Angela Casini, Frank Biedermann, Guillermo Moreno-Alcántar, Pierre Picchetti

**Affiliations:** † Institute of Nanotechnology (INT), Karlsruhe Institute of Technology (KIT), 76344 Eggenstein-Leopoldshafen, Germany; ‡ School of Natural Sciences, Department of Chemistry, Chair of Medicinal and Bioinorganic Chemistry, Technical University of Munich (TUM), 85748 Garching b. München, Germany

**Keywords:** cucurbit[*n*]uril, metal complex, chemosensor, voltammetry, biofluids, drugs, supramolecular chemistry

## Abstract

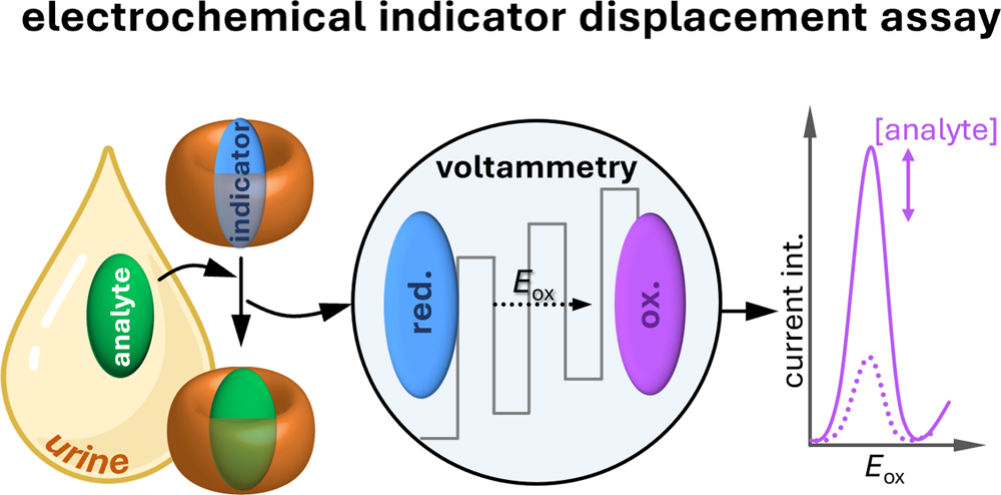

Electrochemical detection methods are attractive for developing miniaturized, disposable, and portable sensors for molecular diagnostics. In this article, we present a cucurbit[7]uril-based chemosensor with an electrochemical signal readout for the micromolar detection of the muscle relaxant pancuronium bromide in buffer and human urine. This is possible through a competitive binding assay using a chemosensor ensemble consisting of cucurbit[7]uril as the host and an electrochemically active platinum(II) compound as the guest indicator. The electrochemical properties of the indicator are strongly modulated depending on the complexation state, a feature that is exploited to establish a functional chemosensor. Our design avoids cumbersome immobilization approaches on electrode surfaces, which are associated with practical and conceptual drawbacks. Moreover, it can be used with commercially available screen-printed electrodes that require minimal sample volume. The design principle presented here can be applied to other cucurbit[*n*]uril-based chemosensors, providing an alternative to fluorescence-based assays.

An important goal for future molecular diagnostic tools is the development of simple, inexpensive, and fast-functioning sensors that can be used at the point of care and operated by nonspecialists.^1,2^ In this context, host-guest chemosensors are considered promising candidates to accomplish this ambitious goal.^3^ At their most basic, chemosensors are macrocyclic molecules (hosts) or host-dye complexes that produce a spectroscopic readout, such as a change in absorbance or fluorescence, after binding to the analyte (guest).^4^ Among the macrocyclic receptors that are known to bind small organic molecules in aqueous media, such as naphtotubes,^5,6^ cavitands,^7−9^ and calix[*n*]arenes,^10,11^ cucurbit[*n*]uril^12^ (CB*n*) receptors exhibit exceptional binding affinity for many biomolecules and drugs (*K*_a_ ≈ 10^3^–10^9^ M^–1^).^13−16^ Furthermore, CB*n* are chemically stable^17^ and biocompatible,^18^ and their synthesis is cost-efficient.

By virtue of their binding affinities, CB*n* are prominent receptors of fluorescence-based chemosensor assays, i.e., indicator displacement assays,^19^ that are compatible with biofluids (Figure 1, left).^20,21^ However, while useful, fluorescence as the signal output can be a disadvantage if the sample being analyzed contains other fluorescent components or larger particles that scatter light, which results in a suboptimal signal-to-noise ratio.^22^ As one alternative strategy for CB*n*-based sensing that circumvents such limitations, our group has recently described a chemosensor assay where the chemiluminescence of a dioxetane-based indicator has been utilized.^23^

**Figure 1:**
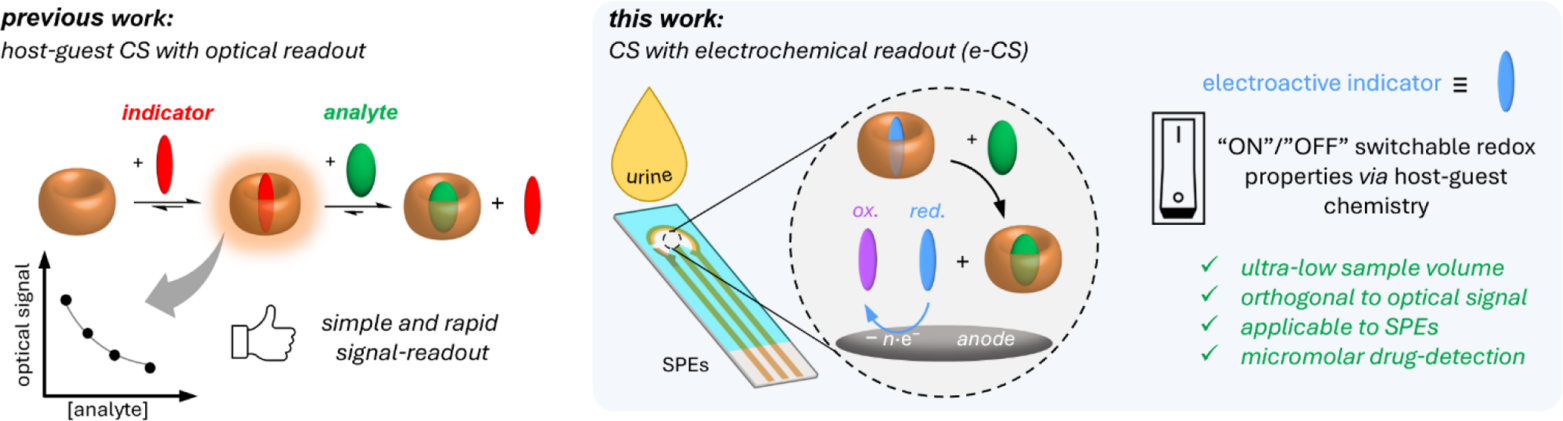
Left: Schematic representation of the working mechanism and features of previously reported optical chemosensors based on host-guest interactions. Right: Schematic representation of the functional principle of the e-CS presented here and its advantageous features.

Electrochemical sensors have emerged as a powerful tool to develop miniaturized, disposable, and portable instruments for molecular diagnostics with relatively inexpensive equipment.^24−26^ In voltammetric or amperometric sensors, the measured signal is a current resulting from direct or indirect electrochemical oxidation or reduction of analytes close to the surface of the working electrode. The commercial success of electrochemical sensors is reflected in their good signal response time to sensitivity ratio and their low-cost components, with commercially available screen-printed electrodes (SPEs) available for sensing purposes that can be further functionalized according to specific needs.^27,28^

Macrocyclic receptors have been used as recognition elements to accumulate electroactive analytes on the surface of electrodes, thereby contributing to an improved signal-to-noise ratio of electrochemical sensors.^29−31^ Such strategies are useful if the analyte can be electrochemically oxidized or reduced within the electrochemical window of water. However, to detect electrochemically inactive analytes through the use of chemosensors, other design strategies are required.

In this regard, the phenomenon that the electrochemical properties of organic molecules can be modulated when they form an inclusion complex with macrocyclic receptors presents an exciting opportunity for the development of chemosensors. For example, it has been shown that the redox current peak for organic molecules decreases significantly when they form an inclusion complex with a macrocyclic receptor.^32−34^ This is because the macrocyclic host can be considered a protective shell surrounding the guest, inhibiting or altering the electrochemical processes or properties of the guest. For example, Ong and Kaifer showed that when ferrocene (Fc) forms an inclusion complex with CB7 (CB7⊃Fc), lower current levels are observed in voltammetric experiments,^35^ which can be attributed to a decreased effective diffusion coefficient of CB7⊃Fc compared to Fc. In addition, a complexation-induced shift in the half-wave potential value for Fc when complexed by CB7 was reported by Kaifer, Kim, Inoue, and co-workers.^32^

As for the competitive guest-displacement from macrocyclic receptors that can be followed electrochemically, Yu and co-workers^34^ have shown in a conceptual example that the oxidation potential of Fc that is immobilized on the surface of gold electrodes (Fc_@surface_) is shifted to more positive values when an inclusion complex with CB7 is formed (CB7⊃Fc_@surface_). By cyclic voltammetry (CV), it was shown that the potential shift indicates the amount of CB7⊃Fc_@surface_ formed in the presence of a competing guest. However, the need for a meticulously uniform self-assembled monolayer on gold electrode surfaces is a major drawback for developing novel electrochemical chemosensors (e-CS). This is because, first, the preparation of monolayers is not straightforward and they have limited electrochemical stability in aqueous solutions.^36^ Second, a monolayer on the electrode surface can lead to nonspecific adsorption of analytes.^34^

In this work, we report a new e-CS assay for detecting the muscle relaxant pancuronium bromide (PB) at micromolar concentrations in buffer and spiked human urine samples (Figure 1, right). The design strategy presented here is based on a competitive binding assay utilizing a chemosensor ensemble that is composed of a new water-soluble and electroactive platinum(II) (Pt(II)) triazole-pyridine complex as the electroactive indicator and CB7 as the macrocyclic host. The indicator exhibits modulated electrochemical properties when it forms an inclusion complex with CB7. However, these properties are restored after its displacement from CB7 and its oxidation is accessible in water and biofluids, e.g., urine. Our e-CS enables the detection of electrochemically inactive analytes in solution and does not require immobilization methodologies on the electrode surface. Therefore, it can be directly used with commercial SPEs.

## Results and Discussion

### Synthesis and Characterization of PtC and Its Host-Guest Complex with CB7

To develop the e-CS, we sought a suitable electrochemically active indicator that can bind to CB7. In fact, although redox-active CB7 guests such as methyl viologen (log *K*_a,CB7_ = 8.8)^37,38^ or [(trimethylamine)methyl]ferrocene^32^ (log *K*_a,CB7_ = 11.5)^33^ exist, their high binding affinity is suboptimal for setting up a functional competitive indicator displacement assay. Indeed, most of the bioanalytes display intermediate affinities (log *K*_a_,_CB7_ ≈ 6), requiring that the indicator should ideally have average binding affinities with CB7 in the range of log *K*_a,CB7_ ≈ 4–7.^38^

**Figure 2:**
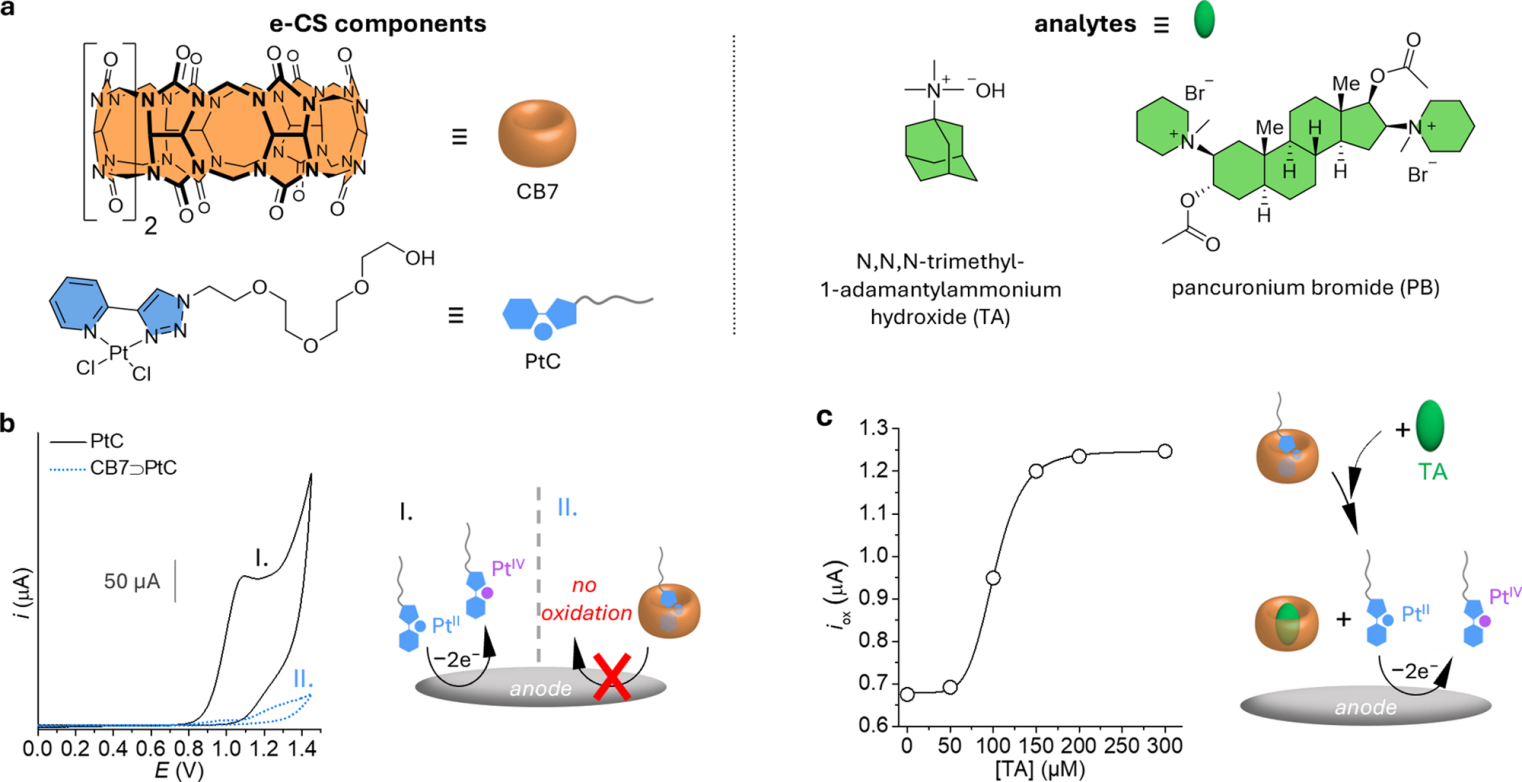
(a) Chemical structures of the macrocyclic receptor CB7, the electrochemically active indicator PtC, and the CB7-binding analytes TA and PB. (b) CV studies of PtC (200 μM) and CB7⊃PtC (200 μM) in water at pH 7.0 (scan rate: 50 mV·s^–1^). (c) TA-dependent *i*_ox_ (at 0.9 V) obtained from CV experiments in water at pH 7.0; [CB7⊃PtC] = 200 μM; scan rate: 50 mV·s^–1^.

In our search for a suitable redox indicator, we identified Pt(II) complexes featuring 2-(1-R-1H-1,2,3-triazole-4-yl)-pyridine ligands as interesting candidates for the following reasons: First, the electrochemical oxidation of the Pt^II^ metal center of the complex to Pt(III/IV) is possible, avoiding auto-oxidation of water. Second, the triazole-pyridine ligand is sterically less demanding compared to other common Pt(II) ligands such as terpyridines and porphyrins, which in turn should enable the formation of an inclusion complex with CB7. Specifically, we synthesized a new triazole-pyridine bearing Pt(II) complex, PtC ((2-(2-(2-(2-(4-(pyridine-2-yl)-1H-1,2,3-triazole-1-yl)ethoxy)ethoxy)ethoxy)ethan-1-ol)dichloroplatinum(II); Figure 2a), which serves as our redox active indicator (see Experimental Section and Figure S1). Briefly, the triazole-pyridine ligand (1) was prepared through a copper-catalyzed azide-alkyne cycloaddition reaction between 2-ethynylpyridine and (2-(2-(2-(2-azidoethoxy)ethoxy)ethan-1-ol) (2) in 62% yield.^39^ The subsequent complexation of 1 using *cis*-dichlorobis(dimethylsulfoxide)platinum(II) as the Pt(II) source gave our final PtC in a very good yield (87%). In our design, the redox chemistry and hydrophobicity of the Pt(II) complex were exploited to provide the required electrochemical redox activity and affinity for the host CB7, respectively, while the hydrophilic polyethylene glycol tail ensures its good water solubility. The photophysical properties of the PtC were next investigated in water. The UV–vis absorption spectrum of the complex (Figure S2) shows a characteristic absorption band at *λ*_max,ab_ = 295 nm, which can be assigned to spin-allowed and ligand-centered transitions, while the absorption occurring at *λ*_ab_ = 310–380 nm is due to a transition with mixed metal-to-ligand charge transfer/ligand-centered character.^39,40^ In water, PtC is weakly blue-emissive (*λ*_ex_ = 300 nm, *λ*_em,max_ = 400 nm, PLQY < 1%), which is due to low-lying metal-centered excited states that are subject to efficient nonradiative decay (Figure S2).^41,42^

The photophysical properties change strongly in the presence of an equimolar amount of CB7 (Figure S3). In particular, the addition of PtC (50 μM) to a solution of CB7 (50 μM) leads to a 2.2-fold increase in its emission intensity in water (pH 7), which is accompanied by an observed blue shift in the emission wavelength maximum (Δ*λ* ≈ 23 nm). Both effects can be attributed to the formation of an inclusion complex between PtC and CB7 (CB7⊃PtC), as the metal complex is protected from the polar solvent and can adapt a more rigid conformation inside the cavity of CB7.^43^ The blue shift in the emission wavelength also suggests that the formation of CB7⊃PtC drives the disaggregation of PtC assemblies that are present in water as well and are formed due to the amphiphilic nature of PtC.

Taking advantage of the observed fluorescence enhancement, we determined the apparent binding affinity value log *K*_a_ = 4.2 for the complex formation of PtC for CB7 in water at pH 7.0 (Figure S4; see Supporting Information). Furthermore, proton nuclear magnetic resonance (^1^H NMR) studies confirmed the binding of the PtC headgroup by CB7 (Figure S5). Contrary to the high binding affinity of other electrochemically active indicators with CB7, the intermediate binding affinity of PtC is advantageous for its use in displacement assays for the detection of bioanalytes.

CV studies further support the formation of the CB7⊃PtC inclusion complex. As shown in Figure 2b, the anodic peak current intensity (*i*_ox_, from 0.9 to 1 V) for CB7⊃PtC is strongly reduced (25-fold) when compared to the free PtC, which can be explained by the shielding effect of the macrocycle preventing the oxidation of Pt(II).^44^ Thus, the formation of the inclusion complex of PtC switches its electrochemical redox behavior to its ″OFF″ state while it can be oxidized in its noncomplexed state, representing its ″ON″ state. With this in mind, we next tested whether this host-guest-mediated modulation in the electrochemical properties of PtC could be switched in the presence of a competitive and high-affinity CB7 guest, i.e., *N*,*N*,*N*-trimethyl-1-adamantylammonium hydroxide (TA; log *K*_a,CB7_ = 12.2; Figure 2a).^45^ We hypothesized that TA would displace PtC from the cavity of the macrocycle and switch its electrochemical ″OFF″ state to the ″ON″ state, which is expected to be reflected by an increasing *i*_ox_. To our gratification, the addition of TA to a solution containing CB7⊃PtC led to a clear increase in the *i*_ox_, as was observed by CV measurements (Figure 2c), until the equivalence point was reached, where all of the PtC was displaced from CB7.

### Electrochemical Detection of Pancuronium Bromide (PB) in Buffer and Real Urine Samples

Pancuronium bromide (Figure 2a) is a steroid-based muscle relaxant used in clinics and general anesthesia. Its extensive use is not without controversy: its improper administration has resulted in cases of nonfunctional anesthesia and has been used by criminals to immobilize their victims;^47^ it is also one of the three components administered for lethal injections in the United States.^48,49^ Under normal anesthesia, PB concentrations in the blood range from 0.3 to 0.5 μM,^50^ whereas PB concentrations in the urine of patients after PB treatment (8 h) were approx. 2.4 μM.^51^ In cases of overdosing, concentrations of up to 2 mM were found in blood and urine.^52^ The detection of PB in pharmaceutical formulations, illicit preparations, and biosamples is mainly limited to mass spectrometry analysis combined with liquid chromatographic techniques,^46,51,52^ as the lack of reactive functional groups in PB and the absence of chromophore units prevented the development of direct or reactive probe-based PB assays. Thus, developing a chemosensor-based, simple, and rapid detection method for PB represents an interesting opportunity to test our e-CS.

**Figure 3:**
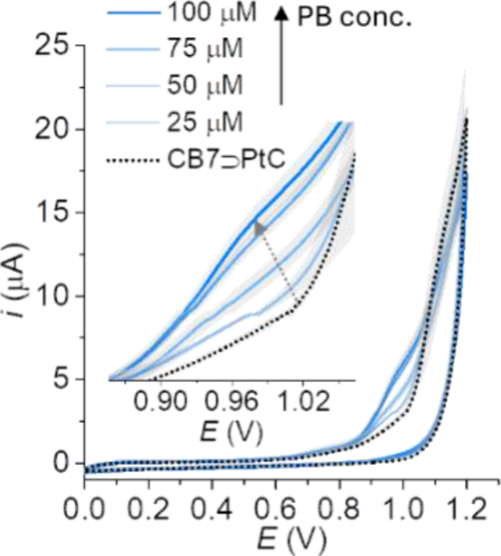
Cyclic voltammetry of CB7⊃PtC (50 μM) in PBS (5 mM, pH 7.0) with and without increasing amounts of PB. The average anodic current and the corresponding standard deviation (*σ*) were calculated from three independent measurements; scan rate: 50 mV·s^–1^.

**Figure 4:**
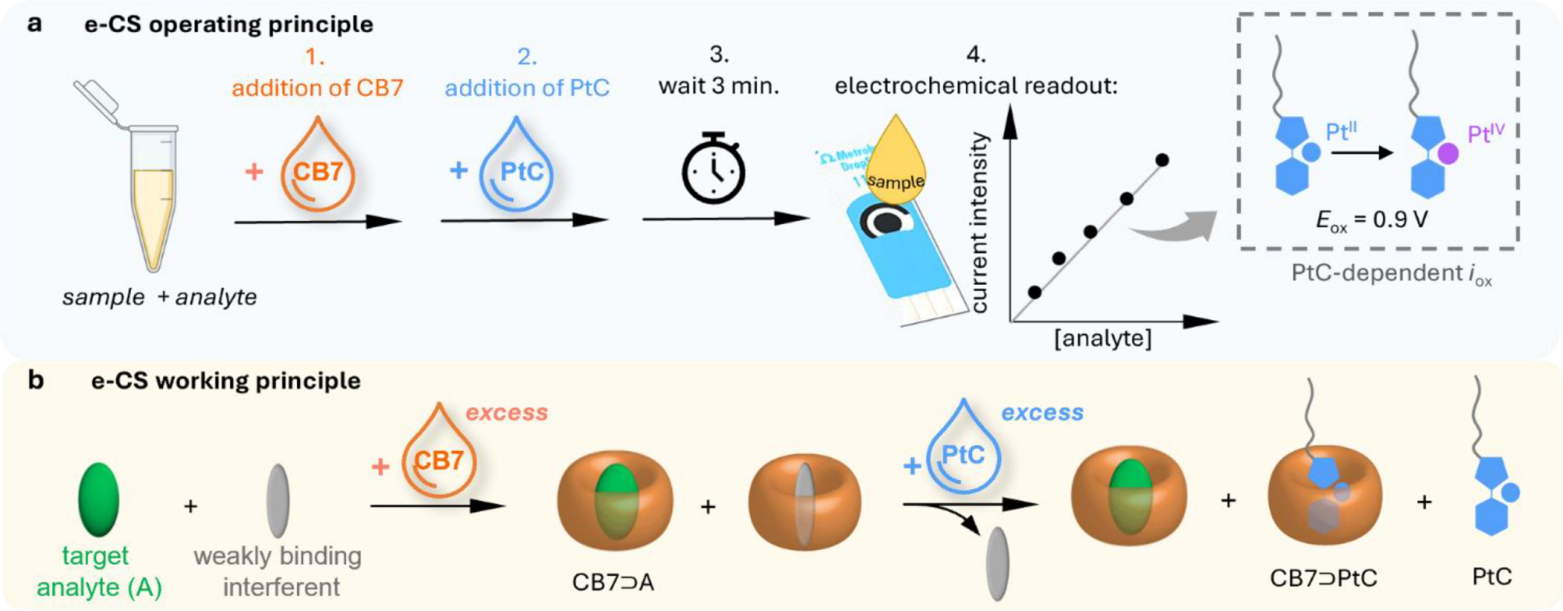
(a) Schematic representation of the operating principle of the e-CS ([CB7] = 50 μM; [PtC] = 50 μM. The electrochemical readout is recorded at a constant *E*_ox_ = 0.9 V. (b) Schematic representation of the working principle of e-CS.

We first examined the CV curves of CB7⊃PtC (50 μM) in PBS (5 mM, pH 7.0) with and without the addition of PB. As shown in Figure 3, a PB-dependent increase in the *i*_ox_ was observed from 0.9 to 1.1 V, which can be explained by the binding of PB to CB7 (log *K*_a,CB7_ = 10.2),^53^ thereby competitively displacing PtC from the cavity of CB7. PB alone shows no significant *i*_ox_ (at 0.9 V is 0.8 μA; Figure S6) since it cannot be oxidized in the potential range studied. To reach higher sensitivity, we adapted the e-CS assay format to chronoamperometric detection (see the Experimental Section). In a typical e-CS experiment (Figure 4a), CB7 (50 μM) and PtC (50 μM) were added sequentially to PB spiked solutions, mixed, and drop-cast (40 μL) onto the SPE. The current was recorded by applying a constant oxidation potential (*E*_ox_ = 0.9 V). The working principle of the e-CS is shown in Figure 4b. PB binds competitively to CB7, thereby releasing PtC from the CB7 cavity, which in turn can be detected by an increase in *i*_ox_ and thus an increase in anodic current density (*j*_ox_).

**Figure 5:**
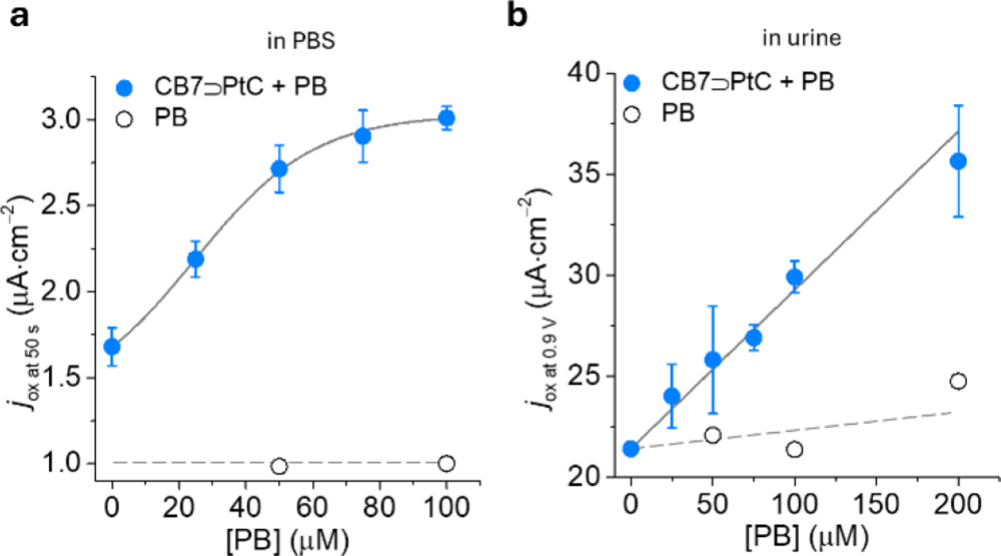
(a) PB-dependent *j*_ox_ (at 50 s) in PBS (5 mM, pH 7.0) with CB7⊃PtC (50 μM) at *E*_ox_ = 0.9 V. (b) SCV-based detection of PB in human urine (1:3 diluted in 5 mM PBS, pH 7.0, [CB7⊃PtC] = 50 μM). Reported is the observed current *j*_ox_ at 0.9 V. The average *j*_ox_ and the corresponding standard deviation (*σ*) were calculated from three independent measurements.

The amperometric response of the e-CS to PB (0–100 μM) in PBS (5 mM, pH 7.0) at 50 s is shown in Figure 5a, and the corresponding chronoamperometric curves are shown in Figure S7a. A PB-dependent increase in *j*_ox_ due to the oxidation of non-complexed PtC is observed, whereas PB alone (Figures 5a and S7b) or the case where PB was mixed with Pt in the absence of CB7 (Figure S7c) caused no change in *j*_ox_. The limit of detection (LOD) was determined to be 17.7 μM (Figure S7d).

Besides chronoamperometry, staircase voltammetry (SCV) or differential pulse voltammetry (DPV) is used in electrochemical biosensors, which offer higher sensitivity, as the occurrence of capacitive charges is diminished through applying a series of regular potential pulse superimposed on the potential stair steps.^54,55^ Therefore, SCV measurements were used to detect PB (0–200 μM) in human urine samples (diluted 1:3 with 5 mM PBS, pH 7.0). As shown in Figure 5b, a PB-dependent increase in *j*_ox_ (at 0.9 V) was observed (Figure S8a), which allowed its detection in PB-spiked human urine. PB itself did not contribute to a significant increase in current (Figure S8b). In the absence of CB7, PB detection is not possible because no change in *j*_ox_ can be observed (Figure S8c). The LOD for PB was calculated to be 7.6 μM in urine, which is sufficiently low compared to the typical concentration levels needed to detect PB misuse or overdose (Figure S8d). Also, DPV measurement yielded a comparable detection limit of 6.3 μM (Figure S9). The adequate amount of CB7 and PtC (50 μM) was selected based on prior concentration screening (Figure S10).

To evaluate the influence of matrix-to-matrix effects on the e-CS, we performed recovery studies using PB-spiked urine samples from two healthy voluntary donors. As shown in Table S1 the e-CS showed good recoveries (>85%) in all the urine samples tested. In addition, we successfully validated our e-CS (Figure S11) with a fluorescence and an LC–MS-based detection method (see the Supporting Information).

### Discussion on the Performance of the e-CS

The e-CS presented in this work operates in solution and can be readily used with commercially available SPEs. Consequently, minimal sample volumes (40 μL) are required and short assay times (6 min) are achieved, which is competitive or superior to optical chemosensor assays.^23^ This represents a considerable advantage as no self-assembled receptor monolayers on the surface of electrodes are required.^34,36,56,57^ Since our assay design does not require surface immobilization, recently reported concepts to improve analyte discrimination by chemosensors, e.g., salt-induced adaptations and the analysis of kinetic binding features, will also apply to the e-CS.^58−60^ The e-CS presented here is the first chemosensor capable of detecting PB via host-guest interactions at low micromolar concentrations (Table 1). Considering the LODs, it can be used for the presumptive detection of illegal or improperly prepared PB formulations and drug overdose detection.^52^ However, the derived LOD values should only be considered approximations since supramolecular host-guest interactions do not follow linear relationships. In addition, it should be noted that the presence of other drugs with high affinity for CB7, e.g., amantadine, negatively affects the detection of PB.^21^

**Table 1:** Comparison of Different Methods for the Detection of PB; n.a., Not Applicable

method	linear range [μM]	LOD [μM]	sample	ref.
enzyme assay	0.3–2.7	0.002	urine, serum	(61)
fluorescence	0.1–1.3	n.a.	extracts of urine, plasma or blood	(62)
HPLC	546.0–1638.0	8.5	pavulon injections	(63)
	68.2–409.4	6.0		(64)
HPLC–MS	0.0–2.7	0.003	extracts of urine, plasma, blood, and gastric content	(65)
potentiometry	9.9–997.3	n.a.	urine and pharmaceutical samples	(66)
voltammetry (this work)	0.0–200.0	6.3	PBS buffer and urine	

## Conclusions

The competitive displacement of an electrochemically active metal complex from CB7 presented in this work is a novel design principle for chemosensor assays that can be used for the electrochemical detection of electrochemically inactive analytes. We have prepared a new Pt(II)-based compound that forms an inclusion complex with CB7 and, after displacement, can be electrochemically oxidized, acting as an indicator in a competitive binding assay format. Furthermore, we demonstrated the detection of the challenging drug pancuronium bromide in aqueous solutions and urine samples. Our e-CS is adaptable to commercially available screen-printed electrodes and operates with minimal sample volumes. Importantly, our design principle circumvents the drawback of previous design principles of other host-guest-based chemosensors with electrochemical readout. We believe that our method can be a useful complement to existing fluorescent chemosensors by providing another route for the development of new sensors, especially for applications at the emergency site where rapid and predictive detection is of importance.

## Supporting Information Available

Details about instruments, materials, and methods, binding affinity studies, supporting figures, and chemical synthesis. The host-guest binding parameters have been deposited at suprabank.org. The voltammetric and amperometric current intensities have been deposited at the RADAR4KIT repository (URL: https://radar.kit.edu/; DOI: 10.35097/862 and DOI: 10.35097/1016). Synthesis procedures and spectra are also available via the Chemotion repository (see the Supporting Information) (PDF)

## Abbreviations

CB7: cucurbit[7]uril
CV: cyclic voltammetry
DPV: differential pulse voltammetry
*E*_ox_: oxidation potential
e-CS: electrochemical chemosensor
*i*_ox_: anodic peak current intensity
*j*_ox_: anodic current density
LC: liquid chromatography
MS: mass spectrometry
PB: pancuronium bromide
PBS: phosphate buffered saline
PtC: (2-(2-(2-(2-(4-(pyridin-2-yl)-1H-1,2,3-triazol-1-yl)ethoxy)ethoxy)ethoxy)ethan-1-ol)dichloroplatinum(II)
SCV: staircase voltammetry
SPE: screen-printed electrode
