# Supplementary material for: Electrochemical Detection of Drugs via a Supramolecular Cucurbit[7]uril-Based Indicator Displacement Assay
Source: ACS Sens. 2023 Jun 20;8(7):2525–32. doi: 10.1021/acssensors.3c00008 (PMC10391622; doi:10.1021/acssensors.3c00008)
Supplement: Supplementary file 1 — se3c00008_si_001.pdf [file se3c00008_si_001.pdf]

Supporting Information

Electrochemical Detection of Drugs via a Supramolecular Cucurbit[7]uril-Based Indicator Displacement Assay

Nilima Manoj Kumar,[1] Patrick Gruhs,[1] Angela Casini,[2] Frank Biedermann,[1]* Guillermo Moreno-Alcántar,[2]* Pierre Picchetti[1]*

[1] Karlsruhe Institute of Technology (KIT), Institute of Nanotechnology (INT), 76344 Eggenstein-Leopoldshafen, Germany;

[2] Technical University of Munich, School of Natural Sciences, Department of Chemistry, Chair of Medicinal and Bioinorganic Chemistry, 85748 Garching b. München, Germany;

Email: frank.biedermann@kit.edu, pierre.picchetti@kit.edu, g.moreno-alcantar@tum.de;

TABLE OF CONTENT

1. Abbreviations ____ ***4***

2. Instruments ____ ***5***

Nuclear magnetic resonance (NMR) spectroscopy ____ 5

Electrospray ionization mass spectrometry (ESI-MS) ____ 5

UV-Vis absorption spectroscopy ____ 5

Fluorescence spectroscopy ____ 5

Electrochemical measurements ____ 5

LC-MS measurements ____ 6

3. Materials and methods ____ ***6***

4. Ethical approval ____ ***7***

5. Binding affinity studies ____ ***7***

Determination of the CB7⊃PtC binding constant ____ 7

6. Determination of the LOD values ____ ***7***

7. Recovery measurements ____ ***8***

8. e-CS validation studies ____ ***8***

General considerations ____ 8

Fluorescence based validation of the e-CS ____ 8

LC-MS based validation of the e-CS ____ 8

9. Synthesis of PtC ____ ***9***

(2-(2-(2-(2-azidoethoxy)ethoxy)ethoxy)ethan-1-ol) (2) ____ 9

(2-(2-(2-(2-(4-(pyridine-2-yl)-1H-1,2,3-triazole-1-yl)ethoxy)ethoxy)ethoxy)ethan-1-ol) (1) ____ 9

(2-(2-(2-(2-(4-(pyridine-2-yl)-1H-1,2,3-triazole-1-yl)ethoxy)ethoxy)ethoxy)ethan-1-ol)dichloroplatinum(II) (PtC). ____ 9

10. Supporting Figures ____ ***10***

Synthesis scheme for the preparation of PtC ____ 10

Absorption and emission spectra of PtC ____ 11

Fluorescence enhancement of PtC in the presence of CB7 ____ 11

Determination of the binding constant of PtC with CB7 ____ 12

NMR-based binding studies of PtC with CB7 ____ 13

CV spectrum of PB in water ______ 14
Chronoamperometric response curves of e-CS for the detection of PB in PBS ______ 15
SCV response curves of e-CS for the detection of PB in urine ______ 16
DPV response curves of e-CS for the detection of PB in urine ______ 17
Optimized amount of CB7⊃PtC ______ 17
Validation of the e-CS ______ 18
NMR spectra, mass spectra and chromatograms ______ 19
11. Supporting Table ______ 24
12. References ______ 24

1. Abbreviations

ACN	Acetonitrile
CB7	Cucurbit[7]uril
CV	Cyclic voltammetry
DPV	Differential pulse voltammetry
ESI	Electrospray ionization
EtOAc	Ethyl acetate
LC	Liquid chromatography
i_{ox}	Anodic peak current intensity
j_{ox}	Anodic current density
λ_{em}	Emission wavelength
λ_{ex}	Excitation wavelength
LOD	Limit of detection
MDAP	2,7-Dimethyldiazapyrenium diiodide
MS	Mass spectrometry
NMR	Nuclear magnetic spectroscopy
PB	Pancuronium bromide
PBS	Phosphate buffered saline
PMMA	Poly(methyl methacrylate)
PtC	(2-(2-(2-(2-(4-(pyridine-2-yl)-1H-1,2,3-triazole-1-yl)ethoxy)ethoxy)ethoxy)ethan-1-ol)dichloroplatinum(II)
rpm	revolutions per minute
SCV	Staircase voltammetry
SPE	Screen-printed electrode
TA	N,N,N-trimethyl-1-adamantylammonium hydroxide

2. Instruments

Nuclear magnetic resonance (NMR) spectroscopy. ^{1}H and ^{13}C NMR spectra were acquired on a Bruker AV400 Ultra Shield or on a Bruker Advance 500 spectrometer at 25 °C. The chemical shifts (δ) are given in ppm and refer to residual protons on the corresponding deuterated solvent.

Electrospray ionization mass spectrometry (ESI-MS). Mass spectra were recorded on a HESI Thermo Scientific Exactive Plus Orbitrap Mass Spectrometer. 5 nM solutions of the compound in LCMS grade acetonitrile were prepared and analysed. The spectra were acquired in positive mode optimized parameters are as follows: capillary temperature: 300 °C, aux gas flux: 2.0 L/min, spray voltage: 4.0 V, spray current 0.36 µA, collision RF: 800 Vpp, transfer time: 120 µs, prepulse storage: 10 µs.

UV-Vis absorption spectroscopy. Absorbance spectra were measured at 25 °C in Milli-Q water on a Jasco V-730 double-beam UV-Vis spectrophotometer. PMMA cuvettes with a light path of 10 mm and dimensions of 10 × 10 mm with a spectroscopic cut-off at 220 nm were utilized for UV-Vis absorption experiments. In addition, the cuvettes were equipped with a magnetic stirrer, allowing rapid mixing.

Fluorescence spectroscopy. Steady-state emission spectra and time-resolved emission profiles for the titration experiments were recorded on a Jasco FP-8300 fluorescence spectrometer equipped with a 450 W Xenon arc lamp, double-grating excitation, and emission monochromators. Emission spectra were corrected for source intensity (lamp and grating) and the emission spectral response (detector and grating) by standard correction curves. All titration experiments were carried out at 25 °C by using a water thermostatic cell holder STR-812, while the cuvettes were equipped with a stirrer allowing rapid mixing. For fluorescence-based titration experiments, PMMA cuvettes with a light path of 10 mm and dimensions of 10 × 10 mm from Brand with a spectroscopic cut-off at 300 nm were utilized.
Fluorescence-based validation experiments were carried out with a CLARIOstar Plus microplate reader using the software-implemented fluorescence method with black isoplates (96-isoplate with black Frame and clear well from Perkin Elmer).

Electrochemical measurements. CV, chronoamperometric, and DPV measurements were performed using a Metrohm portable bipotentiostat/galvanostat (SpectroECL; ± 4 V potential range, ± 40 mA maximum measurable current). The electrochemical setup is controlled by DropView SPELEC software. Screen-printed carbon electrodes (SPEs; DRP-110 from Metrohm DropSense) were used for the measurements. For DPV measurements, the pulse step was 0.02 V, the pulse height was 0.05 V, the pulse duration was 0.1 s, and the sampling rate was 0.02 $V \cdot s^{-1}$. The area of the working electrode is 0.125 cm^2.

LC-MS measurements. LC-MS measurements were performed on an Agilent 1260 Infinity II system consisting of a quaternary pump (GB7111B), autosampler (G7129A, 100 µL sample loop), a temperature-controlled column oven (G7114A) and a variable UV-VIS detector (G7114 A, VWD, flow cell G7114A 018, d = 10 mm, V = 14 µL). Separation was performed on a C18 HPLC-column (Agilent Poroshell 120 EC-C18 4.6 x 100 mm, 2.7 µm) operating at 40 °C. A gradient (refer to gradient table below) of eluent A (ACN + 0.1 vol% Et_3N) and eluent B (Millipore water + 0.1 vol% formic acid) at a flow rate of 1.00 mL·min^{-1} was used. The flow was directed into an Agilent MSD (G6136BA, AP-ESI ion source). The instrument was calibrated in the m/z range 118–2121 in the positive mode and in the range 113–2233 in the negative using a premixed calibration solution (Agilent). The following parameters were used: spray chamber flow: 12 L·min^{-1}; drying gas temperature: 320 K, capillary voltage: 2000 V, fragmentor voltage: 25. The MSD was set up in the singe ion mode (SIM) at m/z = 284.4 at positive polarization.

time [min]	A [%]	B [%]
0.0	80.0	20.0
1.5	80.0	20.0
9.5	0.0	100.0
9.9	0.0	100.0
9.95	80.0	20.0
11.0	80.0	20.0

3. Materials and methods

Tetraethylene glycol (≥ 98.0 %), 2-ethynylpyridine (98.0 %), sodium azide (≥ 99.5 %), p-toluenesulfonyl chloride (≥ 98.0 %), copper(II) sulfate pentahydrate (≥ 98.0 %), (+)-sodium L-ascorbate(≥ 98.0 %), potassium tetrachloroplatinate(II) (98.0 %), acetonitrile (hplc grade), triethylamine (≥ 99.0 %) and formic acid (98.0 %) were purchased from Merck (Sigma-Aldrich). Disodium 4,5,6,7-tetrachloro-2',4',5',7'-tetraiodo-3-oxo-3H-spiro[2-benzofuran-1,9'-xanthene]-3',6'-bis(olate) (rose bengal) was purchased from ThermoFisher Scientific. Pancuronium bromide (> 95 %) was purchased from Cayman Chemical. N,N,N-trimethyl-1-adamantylammonium hydroxide (TA, 25 % in water) was purchased from BLDpharm. Milli-Q water (18.2 MΩ·cm) was collected from the Arium laboratory-grade water purification system. All chemicals were used without further treatment.

CB7 was prepared by following procedures reported in the literature.[1]

[$PtCl_2(DMSO)_2$] was prepared by following procedures reported in the literature.[2]

Stock solutions of CB7, PtC, PB, and TA were prepared in Milli-Q water. The concentration of the CB7 stock solution was determined by fluorescence titration against a known concentration of the MDAP dye (λ_{ex} = 339 nm and λ_{em} = 452 nm) in Milli-Q water. The concentration of stock solutions of CB7 was usually 300 µM in Milli-Q water.

The 5 mM PBS buffer (137 mM NaCl, 2.7 mM KCl, 10 mM Na_2HPO_4, and 1.8 mM KH_2PO_4) was prepared from Gibco PBS tablets and Milli-Q water. The pH of the PBS solution was adjusted to pH 7.0 by adding diluted HCl (in Milli-Q water).

Urine samples were provided by healthy volunteers and were used within 3 – 4 days after excretion (the urine was stored in the fridge at 4 °C). For all the measurements, the collected urine was further diluted (1 : 3) with 5 mM PBS (pH 7.0), and the pH value was adjusted to pH 7.0 by the addition of diluted HCl (in Milli-Q water). The resulting dilution was filtered through a syringe filter (0.45 µm, PES membrane) before use.

4. Ethical approval

All the experiments involving human urine samples were by the formal statement of ethical principles published by the World Medical Association in the declaration of HDeclaration1964 and its later amendments or comparable ethical standards.[3, 4] Informed consent was obtained from all individual participants included in the study.[5, 6]

5. Binding affinity studies

Determination of the CB7⊃PtC binding constant. The binding constant for CB7⊃PtC was determined by a direct displacement assay (DBA), adapting a previously reported procedure.[7] The binding isotherm is obtained by fluorescent titration of PtC (50 µM) with increasing concentration of CB7 stock solution in Milli-Q water at 25 °C (λ_{ex} = 300 nm; λ_{em} = 378 nm). The binding isotherm thus obtained was fitted to a binding model derived from it. S 1 – 4 to obtain the binding constant. All numerical solutions were determined by using the Wolfram Mathematica software (https://github.com/ASDSE/thermosimfit).

$$H + D \leftrightarrows HD \qquad \text{Eq. S1}$$

$$I_{em} = \alpha HD \cdot [HD] + \alpha D \cdot [D] \qquad \text{Eq. S2}$$

$$K_a\,(HD) = [HD] \,/\, [H] \cdot [D] \qquad \text{Eq. S3}$$

$$[D]_0 = [D] + [HD] \qquad \text{Eq. S4}$$

6. Determination of the LOD values

The linear range of the PB-spiked solutions (PBS and urine) curves was fitted with a linear regression curve. The LOD was calculated according to Eq. S5 from three independent measurements.

$$LOD = 3.3 \cdot \left(\frac{\sigma_{jox\ at\ 0.9\ V\ of\ CB7\supset PtC}}{|slope\ of\ linear\ fit|}\right) \qquad \text{Eq. S5}$$

7. Recovery measurements

For the validation experiment, the e-CS calibration curve obtained via SCV measurements was used to analyse different PB-spiked urine samples. The recoveries were calculated by Eq. S6from two independent measurements.

$$\%\,recovery = \left(\frac{[PB]_{observed} - [PB]_{before\,spiking}}{[PB]_{expected}}\right) \cdot 100\% \qquad \text{Eq. S6}$$

8. e-CS validation studies

General considerations. To test the validity of the e-CS, we compared its performance in determining the concentration (DPV mode) of three different PB samples using two complementary detection methods, *i.e.*, a fluorescence-based assay and an HPLC-MS-based assay, which are described below. For this purpose, we prepared PB solutions (in 5 mM PBS, pH = 7) with known concentrations to establish new calibration curves for our e-CS and the fluorescence and LC-MS detection methods. Three different PB solutions were then prepared (15, 30, and 50 µM) and subjected to analysis by e-CS, fluorescence, and LC-MS. The recoveries were calculated according to Eq. S6 and used to validate our chemosensor.

Fluorescence based validation of the e-CS. The fluorescence-based detection of PB was performed following the procedure reported by Poklis and coworkers.[8] Briefly, to the PB-containing sample solution of known concentration (in 5 mM PBS, pH = 7), rose bengal (50 µM; from a 118 µM stock solution in 0.45 M K_2HPO_4) and $CHCl_3$ (300 µL) were added (V_{final} = 600 µL). The resulting mixture was briefly shaken (2 s) and subsequently centrifuged at 8000 rpm for 2 minutes. Subsequently, 200 µL of the $CHCl_3$ layer were carefully transferred to a 96-well isoplate. The fluorescence intensities were measured at 576 nm (λ_{ex} = 546 nm). The calibration curve obtained was used to calculate the PB concentration of the unknown samples (designated as sample 1, 2 and 3) and the recovery values were used to validate the chemosensor. As shown in Fig. S11, both the fluorescence-based and e-CS were able to determine the concentration of the unknown samples with good recoveries.

LC-MS based validation of the e-CS. The LC-MS based detection of PB was performed adapting the procedure reported by Vorce and coworkers.[9] Briefly, the PB-spiked solutions in PBS (5 mM, pH = 7) were used without further treatment and analysed via LC-MS (see Instrument section for further details). As shown in Fig. S11, both the fluorescence-based and e-CS were able to determine the concentration of the unknown samples with good recoveries. The MS spectrum of PB, a representative UV-Vis trace, and a MSD trace used for the validation experiments are shown in Fig. S17.

9. Synthesis of PtC

(2-(2-(2-(2-azidoethoxy)ethoxy)ethoxy)ethan-1-ol) (2). Was synthesized according to the previously reported procedure.[10] ^{1}H NMR (400 MHz, $CDCl_3$) δ 3.76 – 3.72 (m, 2H), 3.70 – 3.66 (m, 10H), 3.66 – 3.59 (m, 2H), 3.40 (t, *J* = 5.0 Hz, 2H). The ^{1}H NMR is in accordance with previous reports.[10]

(2-(2-(2-(2-(4-(pyridine-2-yl)-1H-1,2,3-triazole-1-yl)ethoxy)ethoxy)ethoxy)ethan-1-ol) (1). In a 25 mL round-bottom flask, **2** (2.19 g, 10 mmol), 2-ethynylpyridine (2.06 g, 20 mmol), $CuSO_4{\cdot}5H_2O$ (125.0 mg, 5 mol%), and sodium ascorbate (1.0 g, 20 mol%) were dissolved in DMSO/H_2O (2:1 v/v, 12.0 mL) and the mixture was stirred under N_2 at room temperature for 24 h. The reaction mixture was poured in 100.0 mL of 1 M EDTA in aqueous NH_4OH (25 %) and the mixture was stirred for 1 h. The mixture was extracted with DCM (3 × 50.0 mL), and the combined organics were washed with brine (2 × 50.0 mL) and dried over Na_2SO_4. The organic solvent was removed under reduced pressure. The residue was purified by flash column chromatography (silica gel, 100 % DCM → 5 % MeOH with 1 % NEt_3) to yield a yellowish oil (2.0 g, 62 %). ^{1}H NMR (400 MHz, CD_3CN) δ 8.64 – 8.58 (m, 1H), 8.38 (s, 1H), 8.11 (dt, *J* = 7.9, 1.2 Hz, 1H), 7.86 (td, *J* = 7.8, 1.9 Hz, 1H), 7.31 (ddd, *J* = 7.6, 4.9, 1.3 Hz, 1H), 4.61 (t, *J* = 5.1 Hz, 2H), 3.97 – 3.89 (m, 2H), 3.65 – 3.50 (m, 11H), 3.47 (t, *J* = 4.1 Hz, 2H). ^{13}C NMR (101 MHz, CD_3CN) δ 151.46, 150.50, 148.72, 137.92, 124.18, 123.72, 120.51, 73.22, 71.10, 71.03, 70.94, 70.91, 69.83, 61.86, 51.03. Elemental Analysis (%) Found: C, 55.66; H, 6.91; N, 17.12, Calculated for $C_{15}H_{22}CN_4O_4$: C, 55.89; H, 6.88; N, 17.38. Elemental Analysis (%) Found: C, 55.66; H, 6.91; N, 17.12, Calculated for $C_{15}H_{22}N_4O_4$: C, 55.89; H, 6.88; N, 17.38. HR-ESI $[Na+M]^+$ Calc. 345.1533 Found. 345.1521. See Fig. S10 – 11 for NMR spectra.

See Fig. S12 – 13 for NMR spectra. Additional information on the synthesis and analysis, *e.g.*, full NMR and MS spectra, is available via the Chemotion repository: https://dx.doi.org/10.14272/reaction/SA-FUHFF-UHFFFADPSC-GJRAKKLOML-UHFFFADPSC-NUHFF-NUHFF-NUHFF-ZZZ

(2-(2-(2-(2-(4-(pyridine-2-yl)-1H-1,2,3-triazole-1-yl)ethoxy)ethoxy)ethoxy)ethan-1-ol)dichloroplatinum(II) (PtC). In a 100 mL round-bottom flask, **1** (322.0 mg, 1.0 mmol) and $[PtCl_2(DMSO)_2]$ (422.0 mg, 1.0 mmol) were dissolved in DCM (20.0 mL) and the resulting solution was stirred for 24 h under reflux. After the reaction mixture was cooled down to room temperature, hexane (50.0 mL) was added, and the resulting precipitate was filtered. The obtained solid was redissolved in a minimum amount of DCM and precipitated by adding diethyl ether to yield the compound as a yellow solid (510.0 mg, 87 %). ^{1}H NMR (400 MHz, CD_3CN) δ 9.48 (d, *J* = 5.7 Hz, 1H), 8.76 (s, 1H), 8.21 (td, *J* = 7.8, 1.5 Hz, 1H), 8.04 (d, *J* = 7.7 Hz, 1H), 7.58 (ddd, *J* = 7.5, 5.9, 1.5 Hz, 1H), 4.71 (t, *J* = 5.0 Hz, 2H), 3.97 (t, *J* = 5.0 Hz, 2H), 3.70 – 3.62 (m, 2H), 3.61 – 3.53 (m, 8H), 3.49 (dd, *J* = 5.8, 4.1 Hz, 2H), 2.79 (t, *J* = 5.6 Hz, 1H). ^{13}C NMR (101 MHz, CD_3CN) δ 150.74, 149.81, 149.56, 141.31, 127.14, 126.58, 122.84, 118.26, 73.16, 71.03, 70.85, 70.86, 68.92, 61.81, 53.81. Elemental Analysis (%) Found: C, 30.66; H, 3.52; N, 9.39, Calculated

for $C_{15}H_{22}Cl_2N_4O_4Pt$: C, 30.62; H, 3.77; N, 9.52. Elemental Analysis (%) Found: C, 30.66; H, 3.52; N, 9.39, Calculated for $C_{15}H_{22}Cl_2N_4O_4Pt$: C, 30.62; H, 3.77; N, 9.52. MS (HR-ESI), m/z $[M+Na]^+$: 610.0547.

See Fig. S14 – 15 for NMR and Fig. S16 for MS spectra. Additional information on the synthesis and analysis, *e.g.*, full NMR and MS spectra, is available via the Chemotion repository: https://dx.doi.org/10.14272/reaction/SA-FUHFF-UHFFFADPSC-QQPOZDZNHN-UHFFFADPSC-NUHFF-LUHFF-NUHFF-ZZZ

10. Supporting Figures

Synthesis scheme for the preparation of PtC

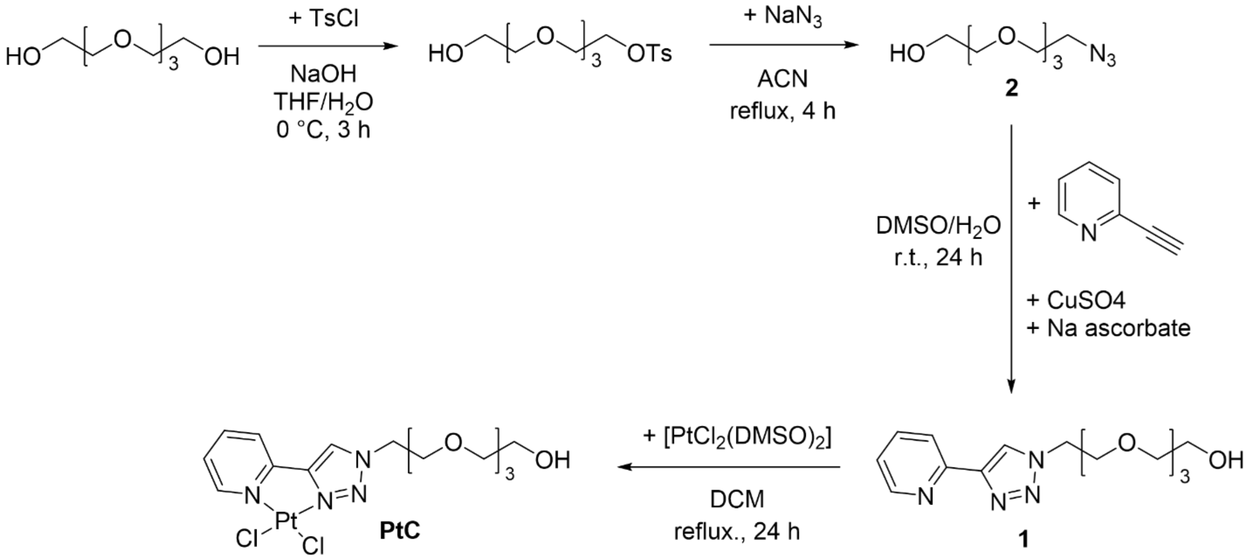

Fig. S1 Reaction Scheme for the synthesis of PtC.

Absorption and emission spectra of PtC

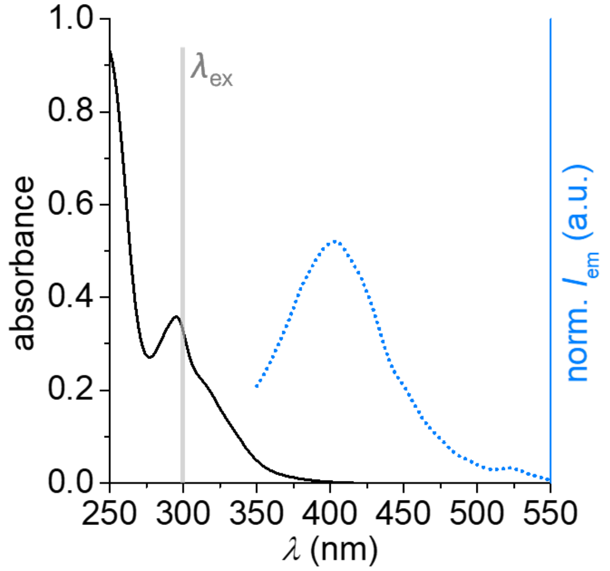

Fig. S2. Absorption and normalized emission spectra (λ_{ex} = 300 nm) of PtC (c = 200 µM) in MilliQ water.

Fluorescence enhancement of PtC in the presence of CB7

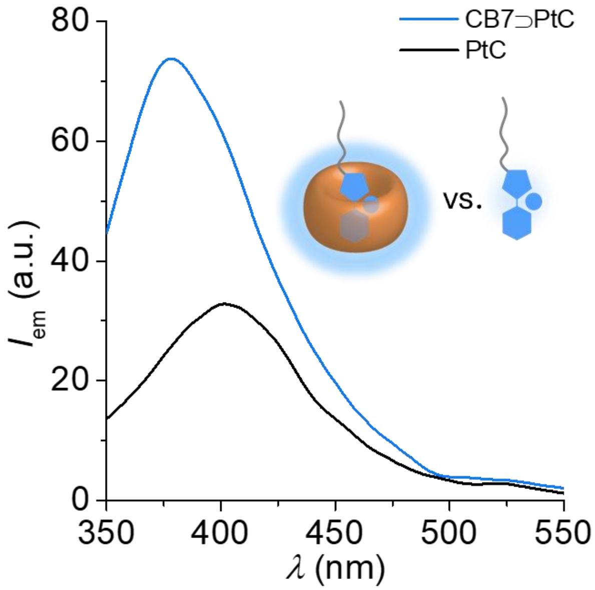

Fig. S3. Fluorescence emission spectra (λ_{ex} = 300 nm) of PtC (c = 50 µM) and CB7⊃PtC (c = 50 µM) in water (pH 7.0).

Determination of the binding constant of PtC with CB7

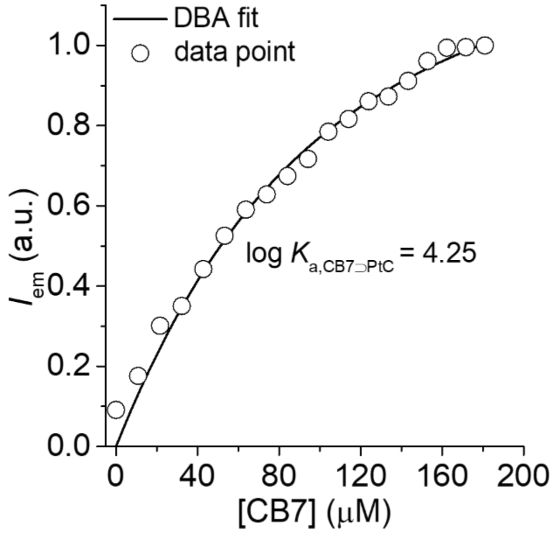

Fig. S4. Representative direct binding assay isotherm determined by the fluorescent titration experiment of PtC (c = 50 μM) with increasing concentration of CB7 in water (pH 7.0) at 25 °C (λ_{ex} = 300 nm, λ_{em} = 378 nm). The estimated fitting error in log K_a is 0.15.

NMR-based binding studies of PtC with CB7

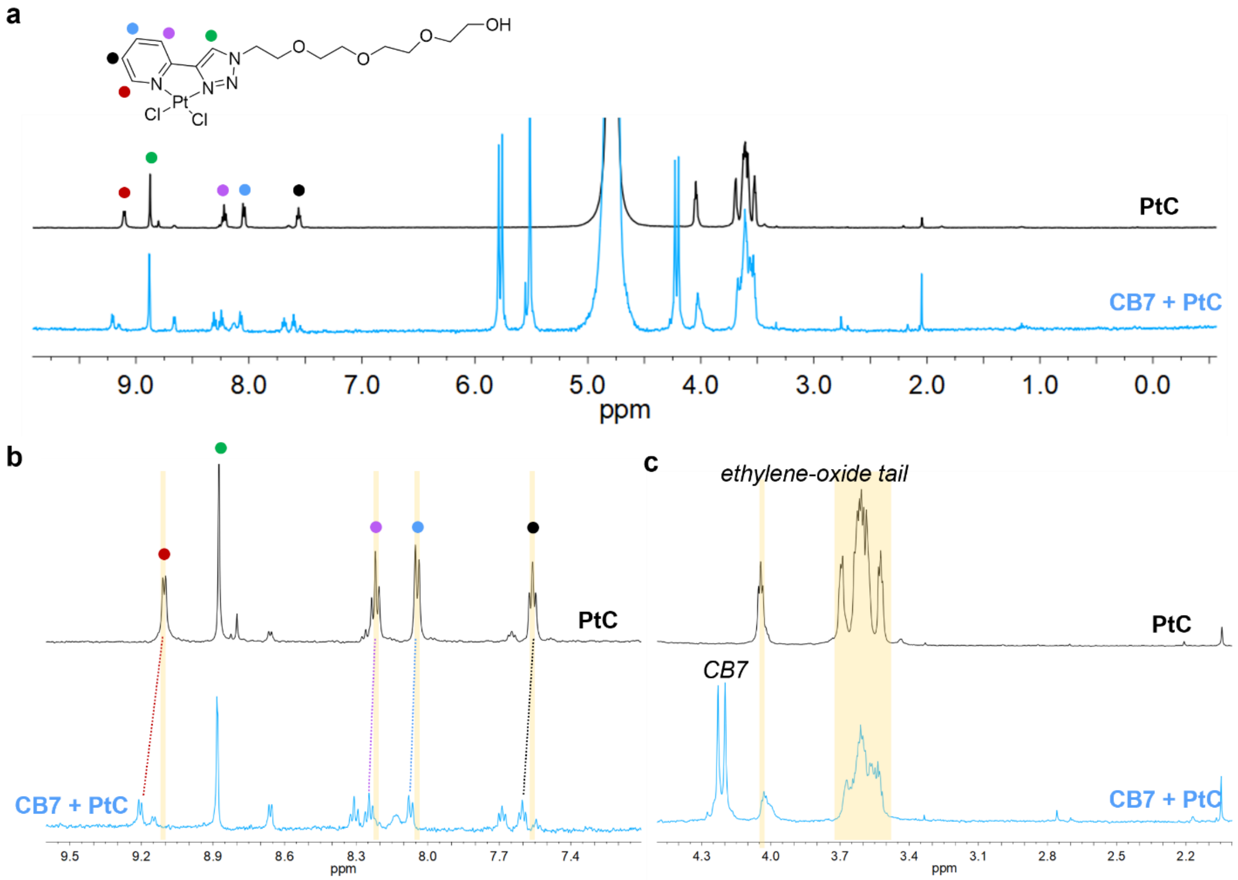

Fig. S5. **a**. ^{1}H-NMR spectrum (500 MHz, D_2O) of PtC (500 µM) and the mixture of PtC and CB7 (both 500 µM). **b** – **c**. Shown are magnified regions of the ^{1}H-NMR spectrum of PtC (500 µM) and the mixture of PtC and CB7 (both 500 µM).

The ^{1}H-NMR spectra allow to determine the geometric features of the CB7⊃PtC complex by following the complexation-induced chemical shifts. At first glance, the observed slight downfield shift of the aromatic proton signals upon addition of equimolar CB7 to PtC (500 µM) is counterintuitive as most reported CB7-guest complexes show upfield shifts of the guests' protons. However, a very similar behaviour was also reported for well-known inclusion complexes of CB7 and acridine orange,[11] thiazine, or methylene blue.[12] Similarly to those organic dyes, amphiphilic PtC has a planar head group that promotes aggregation in solution by head-to-head stacking. Thus the overall observed downfield shift upon CB7 addition results from the de-aggregation of the PtC assemblies, which has a larger effect on the proton shifts than the subsequent PtC binding by CB7.[11]

CV spectrum of PB in water

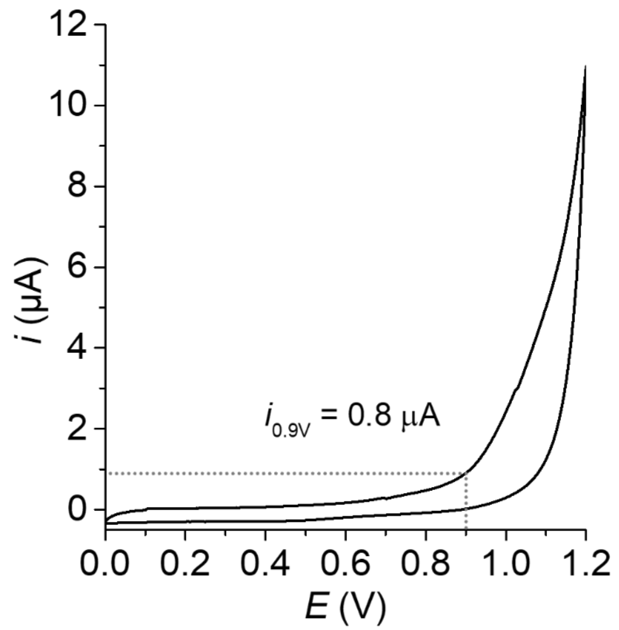

Fig. S6. Cyclic voltammetry spectrum of PB (c = 50 μM) in water (scan rate 50 mV·s^{-1}).

Chronoamperometric response curves of e-CS for the detection of PB in PBS

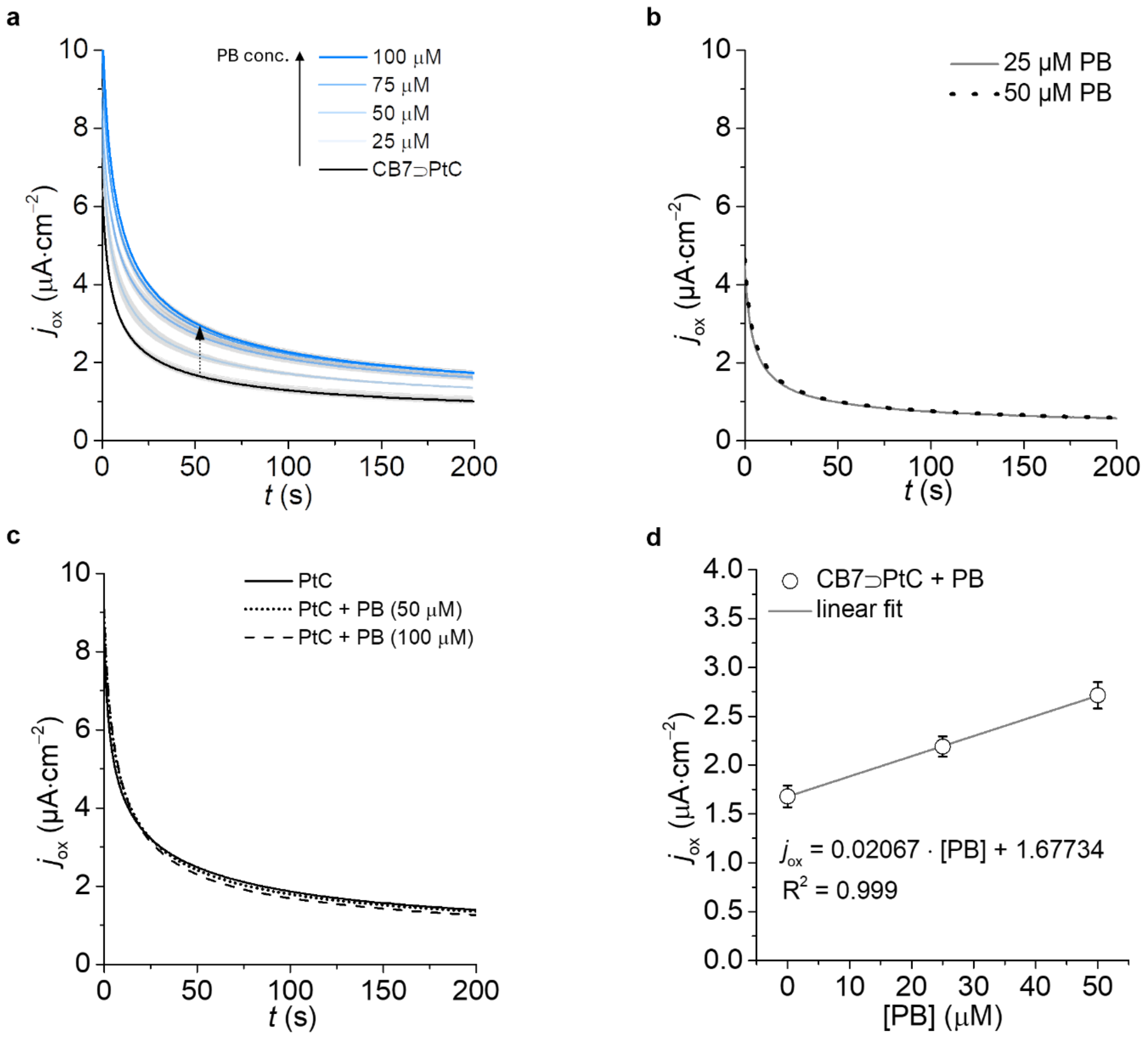

Fig. S7. a. Chronoamperometric response curves for the detection of PB (0 – 100 μM) with CB7⊃PtC (50 μM). The average j_{ox} (coloured lines) and the corresponding standard deviation (σ; grey area) were calculated from three independent measurements. **b.** Chronoamperometric response curves of PB. **c**. Chronoamperometric response curves of PtC (50 μM) without CB7 and in the presence of PB. **d**. The linear regime of the chronoamperometric response curve for the detection of PB was used for the calculation of the LOD. The average j_{ox} and the corresponding standard deviation (σ) were calculated from three independent measurements. All the measurement were performed in PBS (5 mM, pH 7.0).

SCV response curves of e-CS for the detection of PB in urine

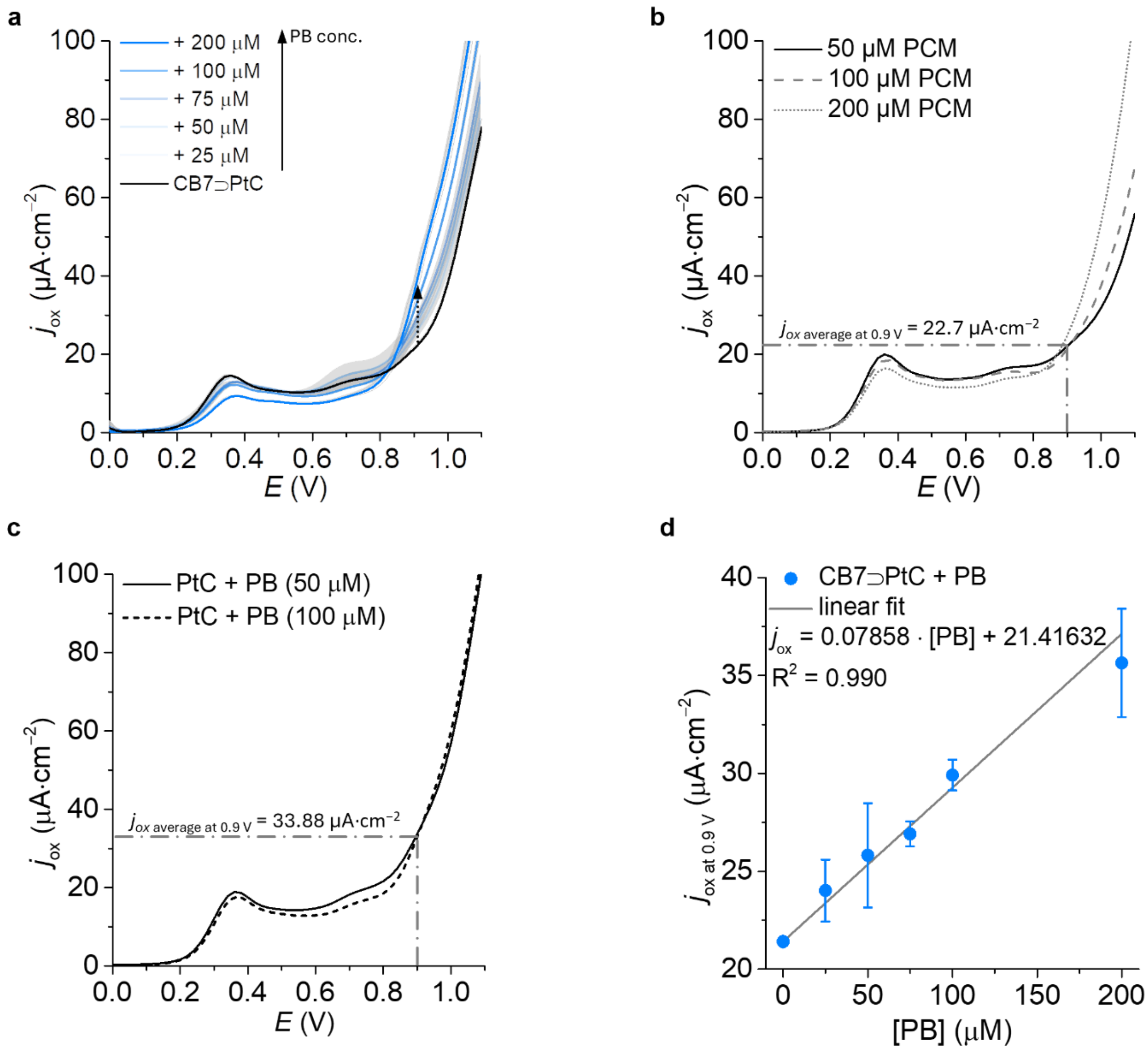

Fig. S8. **a**. SCV curves for the detection of PB (0 – 200 µM) with CB7⊃PtC (50 µM). The average j_{ox} (coloured lines) and the corresponding standard deviation (*σ;* grey areas) were calculated from three independent measurements. **b**. SCV response curves of PB. **c**. SCV response curves of PtC (50 µM) without CB7 and in the presence of PB. **d**. The linear regime of the SCW response curve for the detection of PB was used for the calculation of the LOD. The average j_{ox} and the corresponding standard deviation (*σ*) were calculated from three independent measurements. All the measurements were performed in human urine (1 : 3 diluted with 5 mM PBS, pH 7.0).

DPV response curves of e-CS for the detection of PB in urine

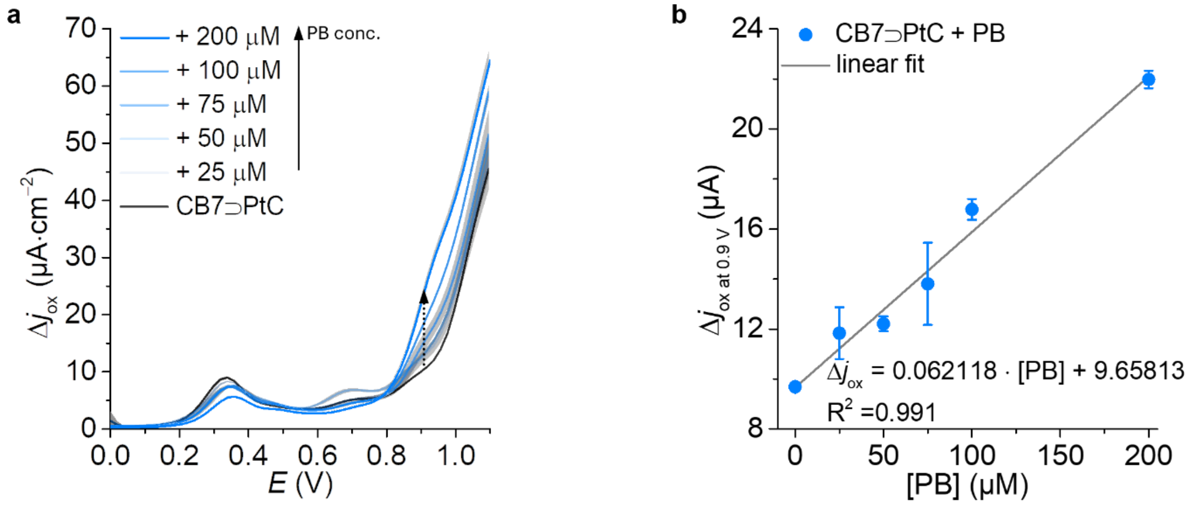

Fig. S9. **a**. DPV response curves for the detection of PB (0 – 200 µM) with CB7⊃PtC (50 µM). **b**. The linear regime of the DPV response curve for the detection of PB. The average Δj_{ox} (coloured lines) and the corresponding standard deviation (*σ;* grey areas) were calculated from three independent measurements. The average Δj_{ox} and the corresponding standard deviation (*σ*) were calculated from three independent measurements. All the measurements were performed in human urine (1 : 3 diluted with 5 mM PBS, pH 7.0).

Optimized amount of CB7⊃PtC

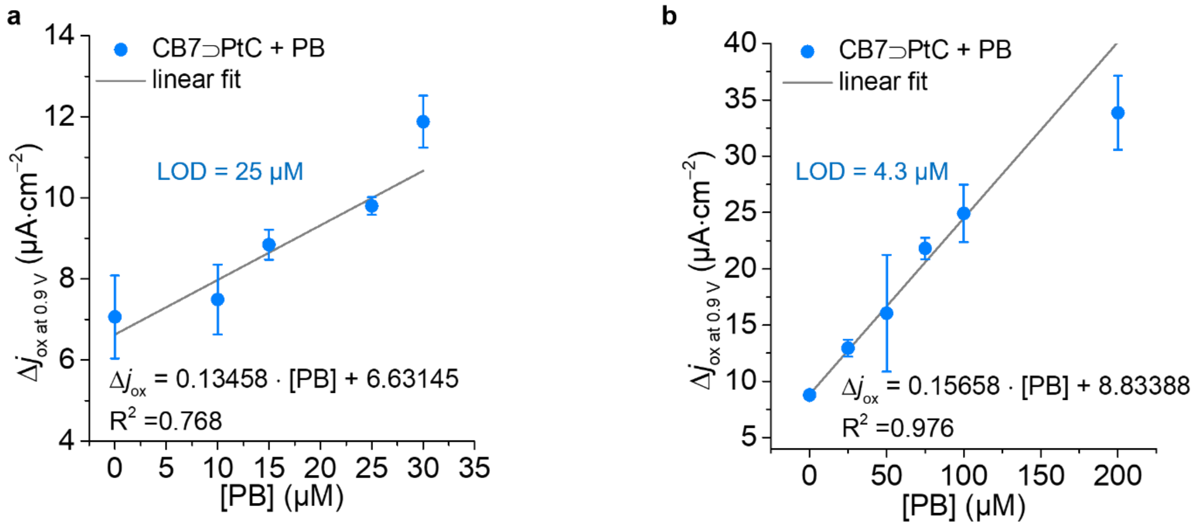

Fig. S10. **a**. DPV response in PBS with 25 µM CB7⊃PtC. **b**. DPV response in PBS with 50 µM CB7⊃PtC. The use of 50 µM CB7⊃PtC represents a concentration at which a satisfactory LOD (4.3 µM) is achieved for the detection of PB. The average Δj_{ox} and the corresponding standard deviation were calculated from three independent measurements.

Validation of the e-CS

a

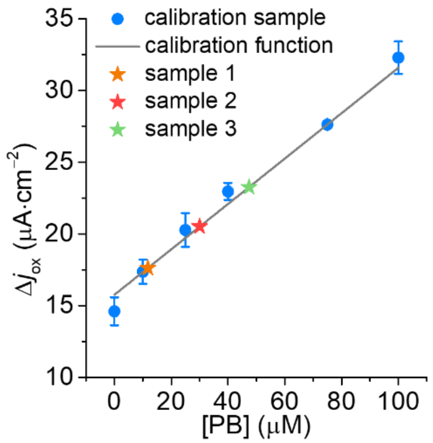

e-CS

results:

sample	spiked conc. [µM]	detected conc. [µM]	recovery
1	15.0	11.8	79 %
2	30.0	30.0	100 %
3	50.0	47.4	95 %

b

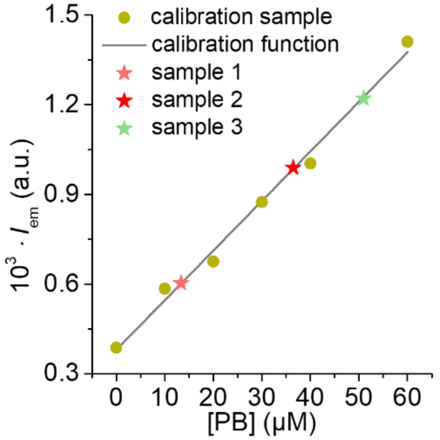

fluorescence analysis

results:

sample	spiked conc. [µM]	detected conc. [µM]	recovery
1	15.0	13.3	89 %
2	30.0	36.4	121 %
3	50.0	50.9	102%

c

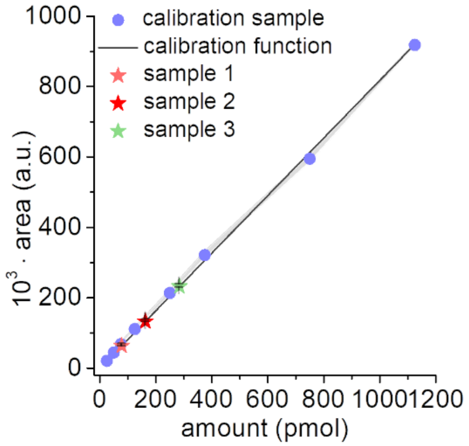

LC-MS analysis

results:

sample	spiked conc. [µM]	detected amount [pmol]	detected conc. [µM]	recovery
1	15.0	78.0 ± 3.8	15.6 ± 0.7	104 ± 4.6 %
2	30.0	162.7 ± 4.0	32.5 ± 0.8	108 ± 2.6 %
3	50.0	284.4 ± 5.5	56.8 ± 1.1	113 ± 2.2 %

Fig. S11. Calibration curve and sample analysis of PB-spiked samples. **a**. e-CS results. **b**. Fluorescence-based detection results. **c**. LC-MS detection method. Errors are calculated from three independent measurements.

^{1}H-NMR spectrum (400 MHz, CD_3CN)

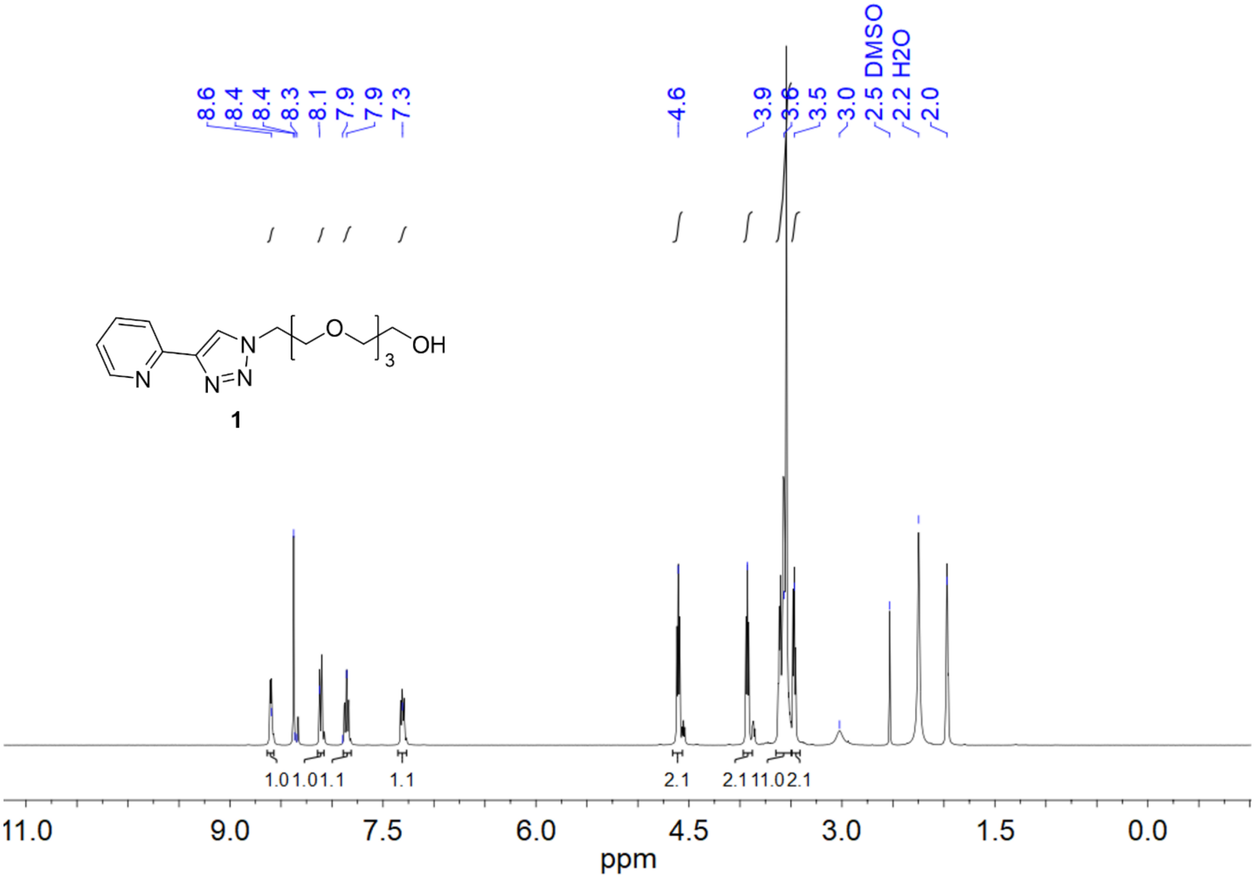

Fig. S12. ^{1}H-NMR spectrum of compound 1.

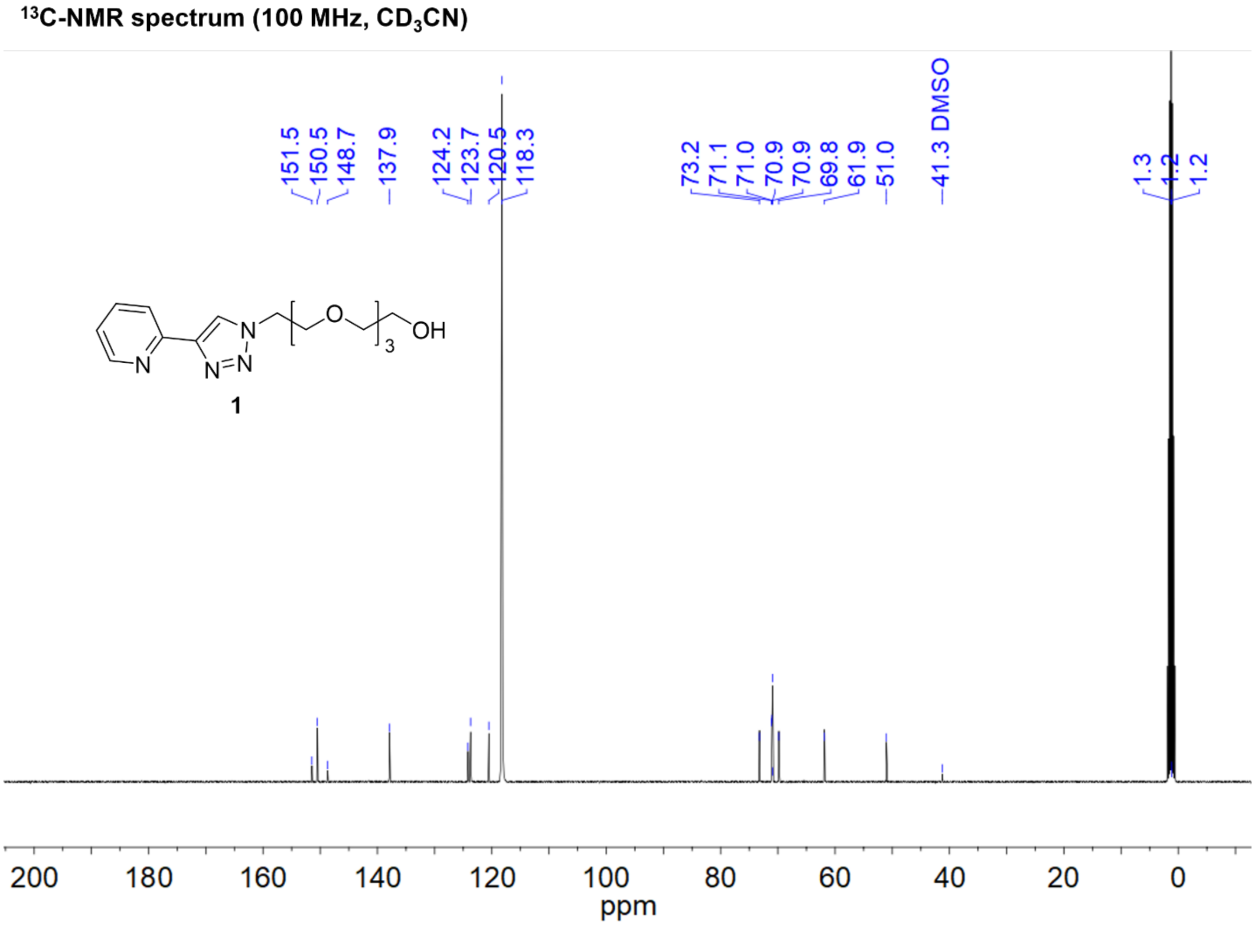

Fig. S13. ^{13}C-NMR Spectrum of compound 1.

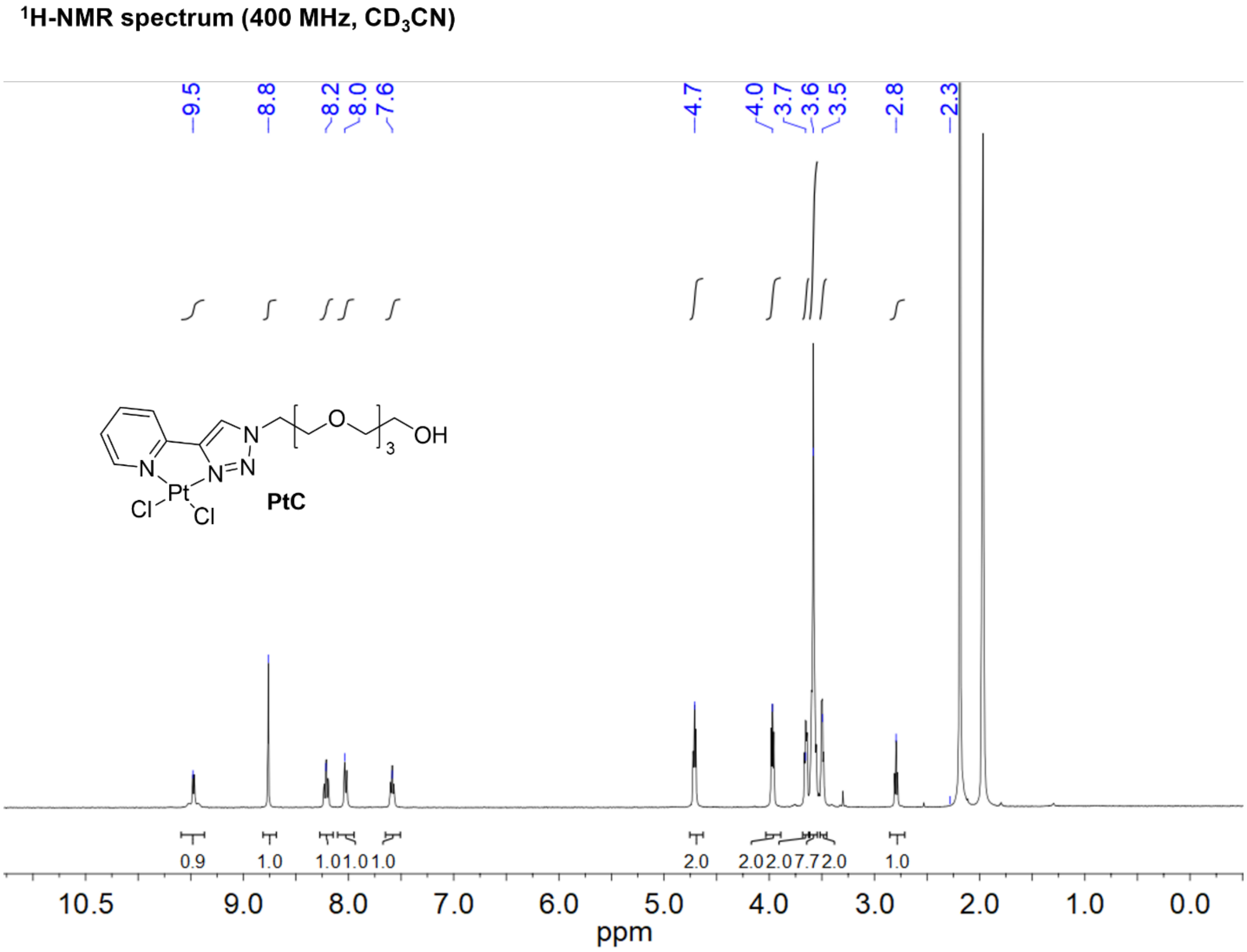

Fig. S14. ^{1}H-NMR spectrum of PtC.

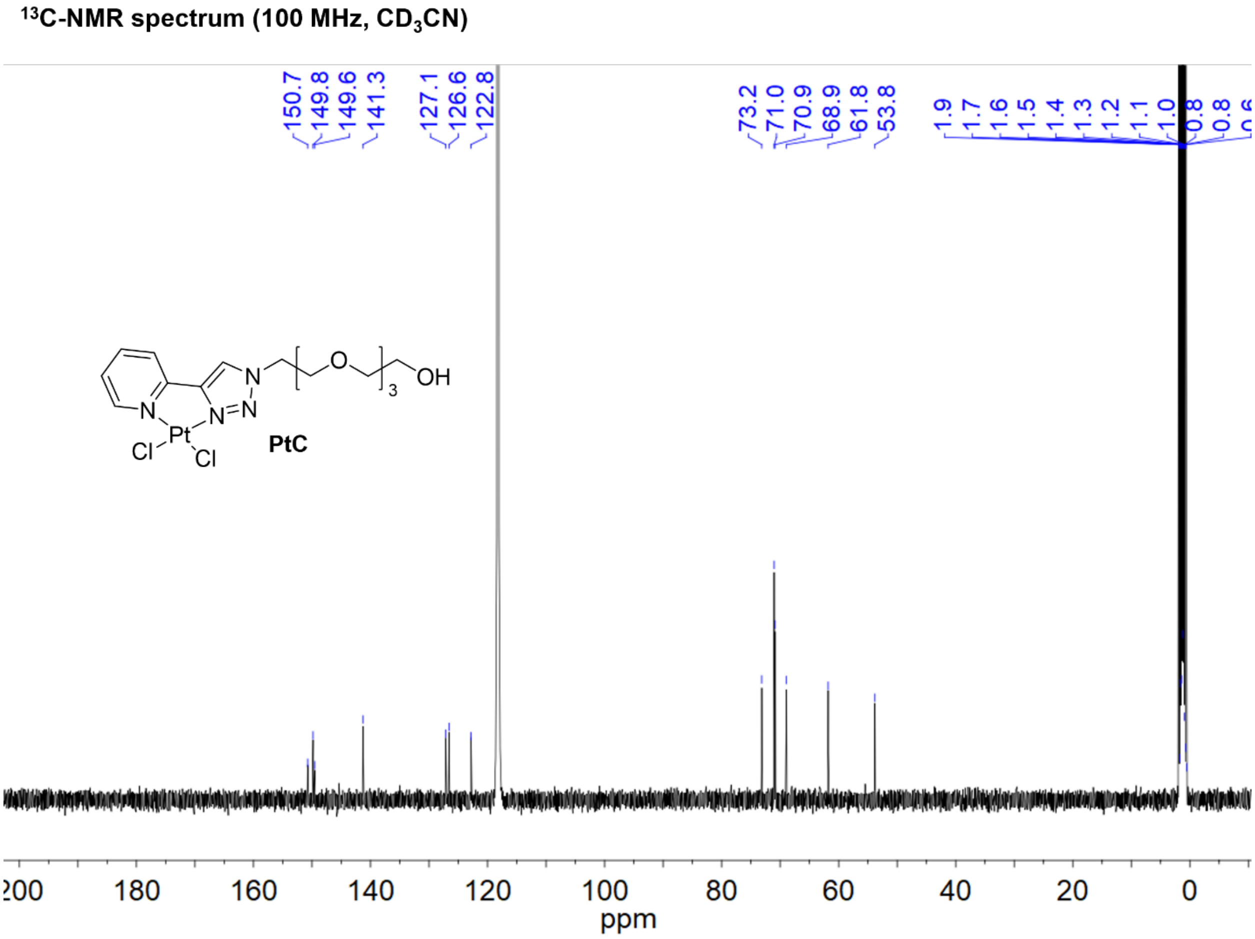

Fig. S15. ^{13}C-NMR spectrum of PtC.

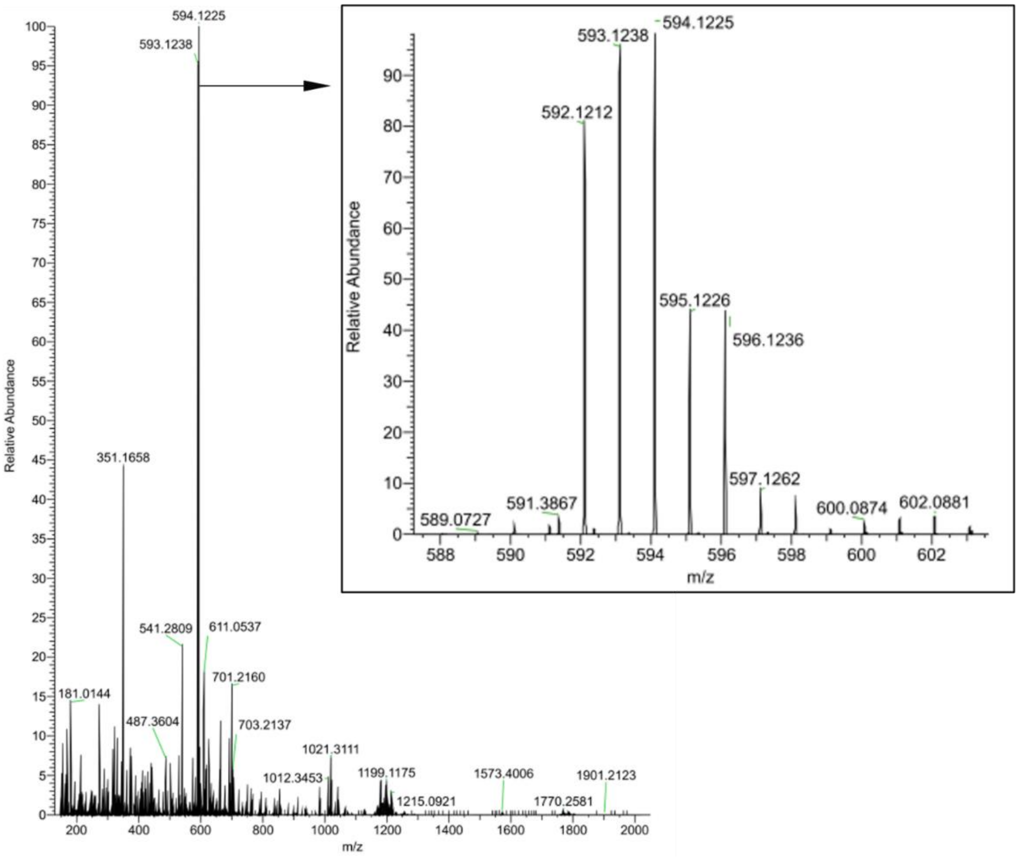

Fig. S16. ESI-MS analysis of PtC.

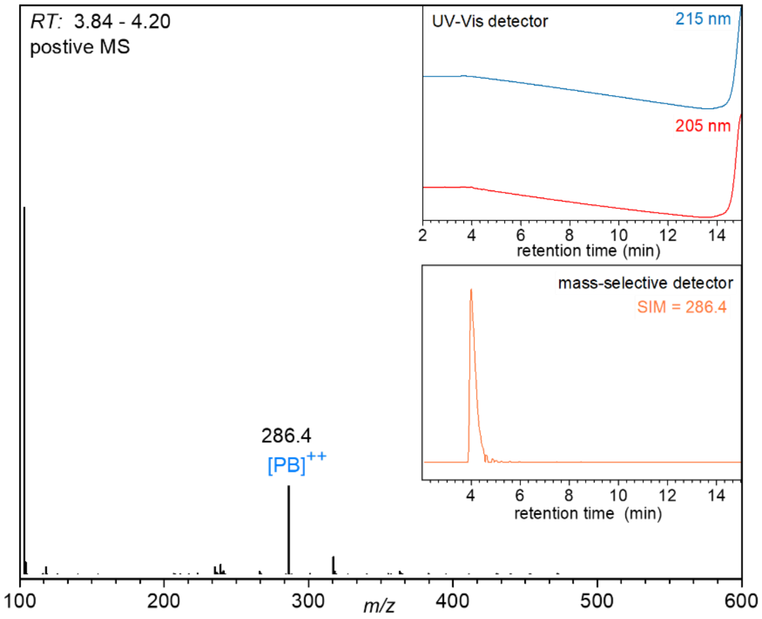

Fig. S17. Positive MS spectrum of PB. Insets: Shown are the UV-Vis traces and the mass selective detector (MSD) response. The MSD response was used to establish the calibration function and detect PB.

11. Supporting Table

spiked conc. [µM]	detected conc. [µM]	recovery
**urine donor 1**		
**5.0**	5.6	112%
**15.0**	13.4	89%
**25.0**	30.4	122%
**35.0**	41.9	120%
**urine donor 2**		
**5.0**	4.8	96%
**15.0**	13.7	91%
**25.0**	29.2	117%
**35.0**	31.0	88%

Table S1. Calculated recoveries for urine-spiked samples from different donors using the e-CS.

12. References

1. Marquez, C.; Huang, F.; Nau, W. M., Cucurbiturils: Molecular Nanocapsules for Time-Resolved Fluorescence Based Assays. *IEEE Trans. Nanobioscience* **2004,** *3* (1), 39-45.
2. Romeo, R.; M., S. L.; Catalano, V.; Achar, S., *Inorg. Synth.* **2007,** *32* (153-158), 153.
3. World Medical Association Declaration of Helsinki: Ethical Principles for Medical Research Involving Human Subjects. *JAMA* **2013,** *310*, 2191-2194.
4. Holm, S., Declaration of Helsinki. International Encyclopedia of Ethics. *Oxford, UK: John Wiley & Sons, Ltd.* **2013**, 1-4.
5. Nijhawan, L. P.; Janodia, M. D.; Muddukrishna, B. S.; Bhat, K. M.; Bairy, K. L.; Udupa, N.; Musmade, P. B., Informed consent: Issues and Challenges. *J. Adv. Pharm. Technol. Res.* **2013,** *4* (3), 134-140.
6. Manti, S.; Licari, A., How to Obtain Informed Consent for Research. *Breathe* **2018,** *14* (2), 145-152.
7. Sinn, S.; Spuling, E.; Bräse, S.; Biedermann, F., Rational Design and Implementation of a Cucurbit[8]uril-Based Indicator-Displacement Assay for Application in Blood Serum. *Chem. Sci.* **2019,** *10* (27), 6584-6593.
8. Poklis, A.; Melanson, E. G., A Suicide by Pancuronium Bromide Injection: Evaluation of the Fluorometric Determination of Pancuronium in Postmortem Blood, Serum and Urine. *J. Anal. Toxicol.* **1980**, *4*, 275-280.
9. Vorce, S. P.; Mallak, C. T.; Jacobs, A, Quantitative Analysis of the Aminosteroidal Non Depolarizing Neuromuscular Blocking Agent Vecuronium by LC-ESI-MS: A Postmortem Investigation. *J. Anal. Toxicol.* **2008**, *32*, 422-427.
10. Moreno-Alcantar, G.; Aliprandi, A.; Rouquette, R.; Pesce, L.; Wurst, K.; Perego, C.; Brüggeller, P.; Pavan, G. M.; De Cola, L., Solvent-Driven Supramolecular Wrapping of Self-Assembled Structures. *Angew. Chem. Int. Ed.* **2021,** *60* (10), 5407-5413.
11. Liu, J.; Jiang, N.; Ma, J.; Du, X., Insight into Unusual Downfield NMR Shifts in the Inclusion Complex of Acridine Orange with Cucurbit[7]uril. *Eur. J. Org.Chem.* **2009,** *2009* (29), 4931-4938.
12. Montes-Navajas, P.; Corma, A.; Garcia, H., Complexation and Fluorescence of Tricyclic Basic Dyes Encapsulated in Cucurbiturils. *Chem. Phys. Chem* **2008,** *9* (5), 713-720.
